# Epigenetic Enhancer Marks and Transcription Factor Binding Influence Vκ Gene Rearrangement in Pre-B Cells and Pro-B Cells

**DOI:** 10.3389/fimmu.2018.02074

**Published:** 2018-09-13

**Authors:** Eden Kleiman, Salvatore Loguercio, Ann J. Feeney

**Affiliations:** ^1^Department of Immunology and Microbiology, The Scripps Research Institute, La Jolla, CA, United States; ^2^Molecular Experimental Medicine, The Scripps Research Institute, La Jolla, CA, United States

**Keywords:** repertoire, enhancer, V(D)J recombination, pro-B cells, pre-B cells, immunoglobulin, Next Generation Sequencing

## Abstract

To date there has not been a study directly comparing relative Igκ rearrangement frequencies obtained from genomic DNA (gDNA) and cDNA and since each approach has potential biases, this is an important issue to clarify. Here we used deep sequencing to compare the unbiased gDNA and RNA Igκ repertoire from the same pre-B cell pool. We find that ~20% of Vκ genes have rearrangement frequencies ≥2-fold up or down in RNA vs. DNA libraries, including many members of the Vκ3, Vκ4, and Vκ6 families. Regression analysis indicates Ikaros and E2A binding are associated with strong promoters. Within the pre-B cell repertoire, we observed that individual Vκ genes rearranged at very different frequencies, and also displayed very different Jκ usage. Regression analysis revealed that the greatly unequal Vκ gene rearrangement frequencies are best predicted by epigenetic marks of enhancers. In particular, the levels of newly arising H3K4me1 peaks associated with many Vκ genes in pre-B cells are most predictive of rearrangement levels. Since H3K4me1 is associated with long range chromatin interactions which are created during locus contraction, our data provides mechanistic insight into unequal rearrangement levels. Comparison of Igκ rearrangements occurring in pro-B cells and pre-B cells from the same mice reveal a pro-B cell bias toward usage of Jκ-distal Vκ genes, particularly Vκ10-96 and Vκ1-135. Regression analysis indicates that PU.1 binding is the highest predictor of Vκ gene rearrangement frequency in pro-B cells. Lastly, the repertoires of iEκ^−/−^ pre-B cells reveal that iEκ actively influences Vκ gene usage, particularly Vκ3 family genes, overlapping with a zone of iEκ-regulated germline transcription. These represent new roles for iEκ in addition to its critical function in promoting overall Igκ rearrangement. Together, this study provides insight into many aspects of Igκ repertoire formation.

## Introduction

The ability of the B-cell receptor to recognize virtually any pathogenic epitope relies on the random nature of Ig V(D)J rearrangement to generate a vast diverse repertoire. Combinatorial diversity through the joining of any V gene with any D or J gene is one of the main contributors to antibody diversity, along with junctional diversity. However, the contribution of combinatorial diversity is an overestimate since individual V genes exhibit great differences in rearrangement frequencies (1–4).

An unbiased method exists for interrogating the RNA repertoire using 5′ RACE PCR to generate a cDNA library with primers at the Cκ or Cμ exon and ligated adaptor (5, 6). Two labs have recently developed an unbiased method for assaying gDNA rearrangements (7–9). Both techniques have their respective advantages and limitations. Use of genomic DNA (gDNA) allows for an unbiased assessment of rearrangement frequencies because each cell only has two chromosomes from which rearrangements will be detected. In contrast, use of RNA examines the repertoire after transcription and could therefore be influenced by differential promoter strengths and post-transcriptional regulation. Also, RNA libraries predominantly assay productive rearrangements due to nonsense-mediated decay of many non-productive rearrangements (10), whereas amplification of gDNA will reveal all non-productive as well as productive rearrangements.

B cell rearrangement in the heavy chain locus and the light chain loci occur sequentially. Heavy chain rearrangements take place first at the pro-B cell stage. The first deep sequencing study of the complete pre-selection Igh repertoire in C57BL/6 pro-B cells using 5′ RACE showed highly uneven V_H_ gene usage across the locus (6). The Igκ locus spans >3 Mb of DNA and contains over 100 functional V genes along with 4 functional Jκ genes (11). 5′ RACE was also used in the first deep sequencing of the Igκ repertoire in bone marrow (BM) B cells. As with the Igh repertoire, this study revealed highly uneven Vκ distribution (5). A recent study by Matheson et al. confirmed uneven Vκ rearrangement frequencies in pre-B cells when assayed from gDNA and they predicted certain transcription factor (TF) binding and epigenetic marks as potentially influencing Vκ gene rearrangement frequency (8).

To date there is no study that has directly and systematically compared repertoires obtained from gDNA and RNA, and since each has potential biases, this is an important issue to clarify. Therefore, in this study, we made libraries from gDNA and RNA from the same batches of sorted small pre-B cells and assessed differences. We found that many Vκ4 family gene members were underrepresented in the RNA repertoire libraries whereas several proximal Vκ3 and Vκ6 family members were overrepresented. Machine learning revealed Ikaros and E2A binding to Vκ gene promoter regions was highly predictive of greater representation in RNA-based libraries, implicating them in creating strong promoters. We found, similar to previous studies (5, 8, 9), that Vκ and Jκ gene usage were very uneven. Using classification analysis with 29 ChIP-seq features and 5 RNA-seq datasets, we show that the RIC score, Ikaros, and PU.1 binding at the RSS best predicted rearranging vs. non-rearranging Vκ genes. Within functional Vκ genes, the levels of newly arising H3K4me1 peaks associated with many Vκ genes in pre-B cells were most predictive of higher pre-B cell gDNA rearrangement levels. Since H3K4me1 is associated with long-range chromatin interactions, which are created during locus contraction, our data provides mechanistic insight into unequal rearrangement levels (12).

It is estimated that roughly 15% of pro-B cells harbor Igκ rearrangements, so we also determined which Vκ genes were the earliest to rearrange by sorting pro-B cells from the same mice as the pre-B cells (13). The pro-B cell repertoire showed an overall bias toward usage of Vκ genes in the Jκ-distal half of the Igκ locus, especially Vκ1-135 and the Vκ10 family genes. Regression analysis showed that different factors regulate Vκ rearrangement in pro-B cells vs. pre-B cells. Lastly, we interrogated the potential role of the kappa intronic enhancer (iEκ) in individual Vκ gene usage in addition to its known role in promoting overall rearrangement levels (14, 15). We show that iEκ^−/−^ pre-B cells display a drastic reduction in both rearrangement and germline transcription (GLT) of Vκ3 family genes. Our data reveals that iEκ diversifies the B cell repertoire by controlling individual Vκ gene rearrangements. Together, this study provides insight into many aspects of Igκ repertoire formation.

## Materials and methods

### Mice

C57BL/6 wild-type and mutant mice were maintained in our breeding colony in accordance with protocols approved by The Scripps Research Institute Institutional Animal Care and Use Committee. iEκ^−/−^ mice were given to us by Dr. Yang Xu (UCSD) (15). Rag1^−/−^ mice were purchased from The Jackson Laboratory (Bar Harbor, ME). We obtained human heavy chain (hIgH) transgenic mice (16) that were bred onto the Rag1^−/−^ background from Dr. Cornelis Murre (UCSD). We generated iEκ^−/−^ Rag1^−/−^ hIgH transgenic mice by breeding iEκ^−/−^ mice with Rag1^−/−^ hIgH transgenic mice.

### Cell sorting

B6 and iEκ ^−/−^ bone marrow (BM) cells were collected from 6- to 7-wk old mice as described previously (6) with the humerus bone collected in addition to the femur, tibia and fibula. CD19^+^ BM B cells were isolated using anti-CD19-coated MACS beads (Miltenyi, Auburn CA).

Each sort used BM from a pool of 3–8 mice. CD19^+^ cells were sorted into pro-B and pre-B cells using a BD FACSAria II at the Scripps Flow Cytometry Core Facility (San Diego, CA). Antibodies are listed in Dataset S1. Pro-B cells were gated as Live^+^, CD19^+^, IgM^−^, CD93^high^, CD2^−^, CD43^+^. Pre-B cells were gated as Live^+^, CD19^+^, IgM^−^, CD93^high^, CD2^+^, CD43^−^. Small pre-B cells were further separated by gating on cell size. Post-sort analysis confirmed purity of B cell fractions. The gating scheme is shown in Figure S1A.

### gDNA and cDNA library preparation for Igκ repertoire deep sequencing

Sorted cells were split into two fractions: one for genomic DNA (gDNA) extraction (DNeasy, Qiagen) and the other for RNA extraction (RNeasy Plus, Qiagen). gDNA libraries were prepared as recently described with several modifications (8) (Figure S1B). gDNA was sonicated to a range of 500–1,000 bp using a Bioruptor sonicator (Diagenode). We omitted the negative depletion step and used different Jκ primers in our protocol (Dataset S1). Library barcoding was performed using NEBNext Multiplex Oligos for Illumina (E7600S).

For RNA, we developed a novel protocol for unbiased cDNA library preparation whereby first strand cDNA synthesis was performed using the Transcriptor High Fidelity cDNA Synthesis Kit (Roche). This was followed by RNase H (NEB) treatment to eliminate RNA complexed as RNA:DNA duplexes. Remaining RNA was eliminated by treatment with RNase A/T1 (Life Technologies). Sample clean-up was performed using Nucleospin Gel and PCR clean-up kit (Machery-Nagel), using NTC buffer to bind ssDNA. Ligation was performed using a 5′ bridge adapter (17) but with an additional 6N added at the 3′ end of the adapter for bioinformatic sequence deduplication. Our cDNA library preparation has two major advantages over the standard technique used for cDNA library preparation for repertoire studies (SMARTer 5′ RACE kit—Clontech). One is that it allows incorporation of random nucleotides to the 3′ end of the oligos (in addition to the 6Ns used for stabilization on the top strand) used for bioinformatic deduplication. Deduplication is the only way to discern whether identical reads, with identical junctional sequences, originated from the same strand of RNA or are an artifact of PCR amplification. Second, the use of a high-fidelity reverse transcriptase enzyme allows for this protocol to be high-fidelity throughout, limiting the number of erroneous nucleotide additions.

After post-adapter ligation cleanup, library preparation was completed via successive PCRs incorporating the NEBNext kit for barcoding. Final library preps were paired-end 2 × 300 sequenced on an Illumina MiSeq System (San Diego, CA) at our Next Generation Sequencing Core. Oligonucleotide sequence and cycling conditions can be found in Dataset S1.

### VJ gene analysis

Demultiplexed paired-end reads were adapter trimmed and quality filtered (Phred>20) using TrimGalore (max of 50 bases trimmed from 3′ end of read, if more bases trimmed from either read then both reads of the pair were thrown out). Paired-end reads were then merged using pandaseq using default settings. VJ gene calling was performed on merged reads using Abstar (https://github.com/briney/abstar) with a custom version of the minimal output setting, appending Vκ gene length in the output. In addition, a Vκ gene reference file was custom made for C57BL/6 (^*^01 alleles) and each Vκ gene was cross-referenced to the mouse genome build mm9 on UCSC genome browser. Abstar output was processed with a custom R pipeline (https://github.com/salvatoreloguercio/RepSeqPipe) developed by S.L. which computed Vκ or Jκ gene usage statistics. The cutoff for Vκ gene assignment was a minimum read length of 150 bp and a minimum of 95% sequence identity. Reads passing this filter were then deduplicated based on the six random adapter nucleotides (gDNA and RNA) and in the case of gDNA, samples were additionally deduplicated based on the starting position of the Vκ gene read which are random due to shearing. Reads that contained the same start site (for gDNA) and random 6Ns were presumed to have originated from the same fragment and only counted once. For gDNA, reads needed further processing to deal with Jκ PCR primer cross-amplification in order to accurately re-assign Jκ gene calling. gDNA reads were re-assigned to their proper Jκ gene based on the sequence upstream of each Jκ primer. This did not include the most Vκ proximal nucleotide of the Jκ exon to allow for potential VκJκ junctional loss. One pre-B cell gDNA library was prepped using a second set of Jκ PCR primers located further downstream of the original Jκ primers (closer to the biotin). Since these Jκ primers were further away from the VκJκ junction, we excluded the 6 most Vκ proximal nucleotides from the Jκ exon in Jκ gene identification.

### Post-pipeline adjustments

We noted that three pairs of Vκ genes were 100% identical at the 3′ most 150 bp of sequence; Vκ5-43 and Vκ5-45, Vκ8-16 and Vκ8-23-1, Vκ13-84 and Vκ13-85. These genes could only be discerned if the read was long enough to include the 5′ end of the gene. This meant that there were reads that passed our Vκ gene length threshold of 150 bp but were assigned to one of the pair arbitrarily. This was more of an issue for gDNA where not all fragments covered the entire gene whereas most RNA transcripts did cover the entire gene. For each of these pairs of Vκ genes, we isolated their reads and performed a search for a sequence string far upstream in the Vκ gene that would discern among the pair. The ratio of this string search was used to re-calculate among the total reads of those two genes within a given sample. More information on this can be found in Dataset S1. We used the corrected orientation for Vκ8-23-1 and corrected Vκ4-60 RSS site recently described (8). In addition, we classified four genes as non-psuedogenes as described by IMGT; Vκ8-18, Vκ1-35, Vκ14-126, and Vκ1-131. Processed read data for different samples is available in Dataset S2.

### ChIP-seq

ChIP-seq was performed as previously described (18). All ChIP-seq data have been deposited in the Gene Expression Omnibus database (Table S1) and uniformly processed following the procedure below.

SRA files obtained from GEO were converted to fastq files using SRA Tools 2.8.2 (*fastq-dump –skip-technical –readids –dumpbase –split-files –clip*). Preliminary quality control over raw sequence data was performed with FastQC 0.11 (19). Duplicate reads were removed before mapping, and TruSeq adapter sequences were removed with the HOMER trim tool (20). Experimental fastq tags were aligned to the mouse reference genome (mm9) using Bowtie 1.1.2 (alignment parameters: *-a -v 2 -m 3 –best –strata*) (21). Confident CTCF and Rad21 peaks were called using MACS (v1.4.2) (22), with a false discovery rate (FDR) ≤ 1% and the default P value (1E-5).

### RNA-seq

RNA-seq was performed as described in Kleiman et al. (18). After quality control as described above for ChIP-seq, raw data were aligned to the mouse reference genome (mm9) using TopHat 2.1.0 and Bowtie 2.2.6 (21, 23). Strand-specific wig files were obtained from alignment (bam) files with IGVTools 2.3.69 (*igvtools count –strands read*) (24).

### Quantification of chromatin and RNA features

For ChIP-seq, alignment (bam) files were first converted to tag directories with HOMER CreateTagDirectory (20). The signal intensity of each chromatin feature for each region was computed with HOMER AnnotatePeaks, where each tag directory was normalized by the total number of mapped tags such that each directory contained 10 million reads (*annotatePeaks.pl mm9 -size given -noann -nogene*) (20). For RNA-seq, signal intensity was calculated from the corresponding wig files (*annotatePeaks.pl mm9 -size given -noann -nogene -wig*). Additional downstream analysis and manipulation of the data, including annotation of peaks, motif finding and overlap analysis, were performed with HOMER 4.7 and R/Bioconductor (25). GEO accession numbers are listed in Table S1.

### Classification and regression models

The dataset includes 162 observations (Vκ genes). To assess feature importance in predicting Vκ gene activity, and magnitude of rearrangement frequency for active genes, we adopted a two-step supervised learning strategy. We first trained a classifier to predict Vκ gene activity, and then built regression models to predict: (1) recombination levels and (2) RNA/gDNA rearrangement ratios of active genes. Relative variable importance was then extracted from the validated classification and regression models. We used Random Forest (RF) for both classification and regression tasks since it handles well high dimensionality (high number of features relative to low number of observations available for training) and feature collinearity, and is robust to overfitting (26).

We divided the read data for each feature into four non-overlapping windows for each Vκ gene: promoter window (500 bp upstream of the start of leader 1 plus leader and its intron); RSS window (Vκ coding region plus 500 bp downstream); upstream window (2.5 kb upstream of the promoter window); downstream window (2.5 kb downstream of the RSS window).

These four windows were computed for each of the 29 ChIP-seq features and 5 RNA-seq datasets (all GEO accessions available in Table S1), giving a total of 132 chromatin and RNA expression features. We also included two genetic features: the RIC score and the distance from the Vκ gene to Jκ1. Thus, a total of 134 features were considered as explanatory variables for both classification and regression tasks. Analysis targeting a pre-B cell response used both pre-B cell and pro-B cell features, whereas analysis of pro-B cell responses used pro-B cell features only.

For classification, we used binary recombination status (inactive/active) as a response variable with a threshold for active VκJκALL genes of 15 reads per million reads yielding 125 active Vκ genes (24 of which were pseudogenes) and 37 inactive Vκ genes in pre-B cell gDNA. VκJκ1 active gene list was derived from the VκJκALL gene list. For regression, the response variable used was the recombination frequency of 125 active Vκ genes, defined as the sum of reads from all biological replicates divided by the total number of reads. The same analysis was performed on pro-B cell gDNA but in this case 1 read was the cut-off for active Vκ genes due to the limited number of reads. Using this threshold, there were 108 active Vκ genes, of which 13 were pseudogenes. For pre-B cell regression analysis on RNA/gDNA ratios, only the active functional Vκ genes were considered.

Both classification and regression were performed with 10-fold cross validation, i.e., 10% of Vκ genes were assigned to the test set each time, with every gene included in a test set exactly once. The number of trees generated for each fold was 5,000. For classification, the number of variables randomly sampled as candidates at each split (*mtry*) was optimized using the *tuneRF* routine from the R package *caret*; default parameters were used for the regression models. The average importance of each feature was recorded.

### Model accuracy

For the classification model, performance was assessed by accuracy, i.e., the percentage of correct predictions across all 10 test sets. Performance of the regression model was assessed by the root mean squared error (RMSE) for the predicted recombination frequencies vs. the observed values across all 10 test sets.

For feature selection, we considered the 20 most important variables from the initial classification or regression models and used Recursive Features Elimination (RFE) (*rfe* in the R package *caret*) to train RF models for all possible combinations of the respective 20 features. Cross-validated (10-fold) prediction performance of models with sequentially reduced number of predictors (ranked by variable importance) was then used to suggest significant predictors. The models were evaluated using the performance metrics described above. All analyses were performed using the R packages *randomForest* (27), *caret* (28), and *mlbench*. Plots were generated with the R packages *ggplot2, gtools, ggpubr* and Prism graph software (La Jolla, CA).

### Data availability

Publicly available and Feeney lab generated genome-wide ChIP-seq and RNA-seq datasets analyzed in this study are available in the GEO repository. GEO accession numbers are listed in Table S1. GEO accession numbers for gDNA and RNA VκJκ-seq datasets generated in this study are also listed in Table S1.

### Rearrangement and GLT qPCR

Pre-B cell gDNA from B6 wild-type and iEκ^−/−^ mice was used for TaqMan qPCR to assay for rearrangements. Primer and probe sequences are listed in Dataset S1. TaqMan Master Mix II (#4440041) was purchased from Applied Biosystems (Foster City, CA). Jκ1 and Eμ ZEN probes were purchased from IDT (San Diego, CA). To assay GLT, pre-B cell RNA from B6 Rag^−/−^ hIgH Tg and iEκ^−/−^ Rag^−/−^ hIgH Tg (7–14 weeks of age) were used for SYBR Green qPCR. GLT primer sequences are listed in Dataset S1. SYBR Green 2x master mix (#21203) was purchased form Biotool (Houston, TX).

### Statistics

Statistical analysis on bar graphs was done using Prism software.

## Results

### VκJκ repertoire reveals unequal Jκ and Vκ usage

We performed Igκ light chain sequencing on 3 pre-B cell gDNA replicates using a modification of VDJ-seq (7, 8) with a strict gating scheme that excluded any IgM^low^ immature B cells (Figures S1A–C). Repertoires from the 3 gDNA preparations were 99% identical (Figure S2A). Pooling the reads from all 3 replicates, we were able to detect 133 Vκ genes with at least one read, 32 of which were classified pseudogenes by IMGT. Dataset S2 summarizes read statistics for all samples, as well as the total number of reads for each Vκ gene. The average ratio of non-productive to productive from the 3 gDNA replicates was 67:33 (Figure S3A), at the expected two-thirds non-productive frequency. The nomenclature that we use is that of IMGT in which the first number is the Vκ family and the number after the dash is its position within the locus, with Vκ genes numbered consecutively from 3-1, the most Jκ-proximal Vκ gene, to 2-137, the most Jκ-distal Vκ gene. A map of the V, J, and C genes can be seen on the IMGT website (http://www.imgt.org/IMGTrepertoire/LocusGenes/#B).

**Figure 1:**
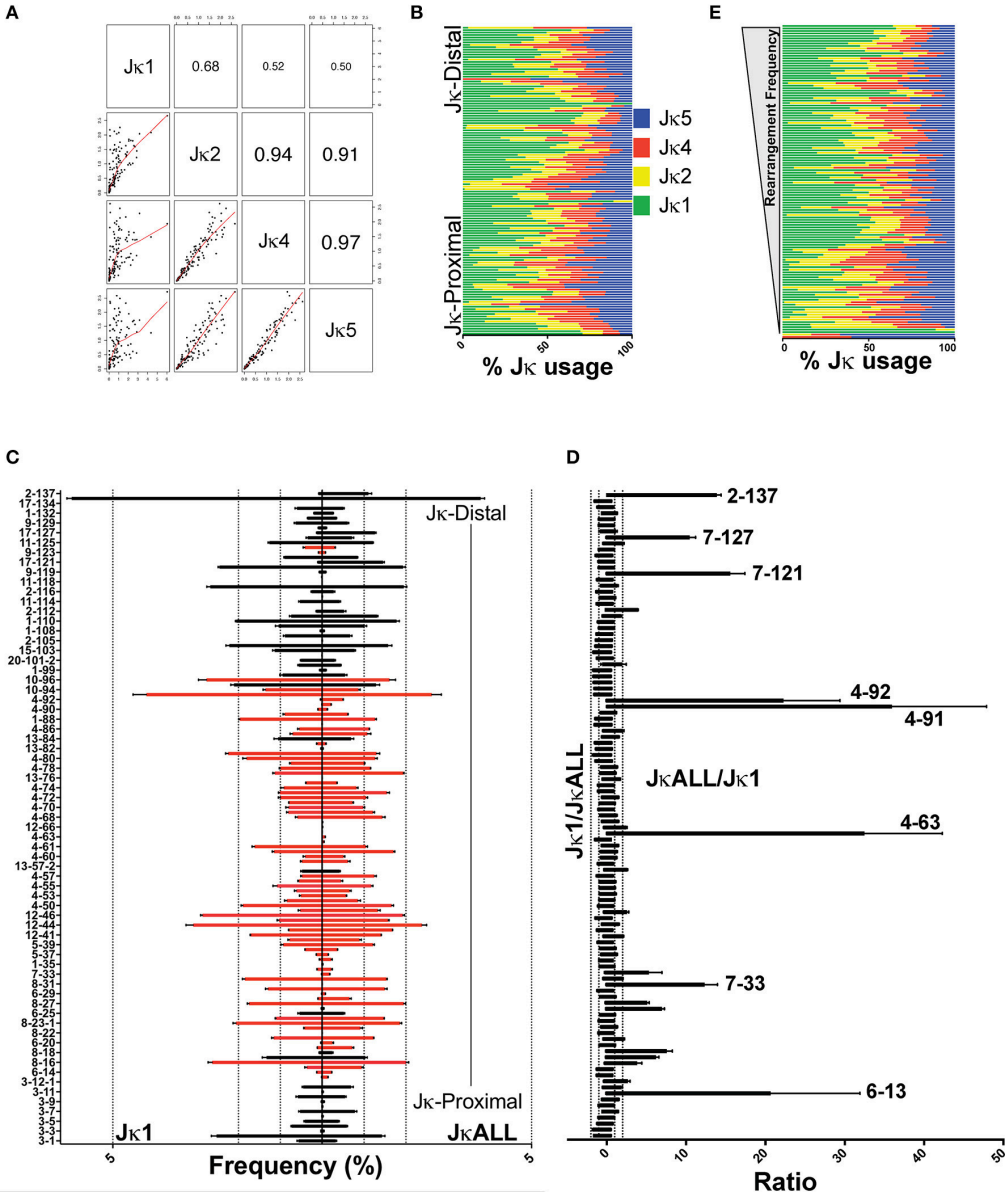
Variation in VκJκ repertoires. **(A)** Scatterplot matrix showing correlation between VκJκ1, VκJκ2, VκJκ4, and VκJκ5 repertoires. Lower left panels depict scatterplot matrices. Upper right panels depict absolute correlation. The font size of the correlation value is proportional to the correlation. **(B)** Percentage of Jκ gene usage for each Vκ gene arranged vertically from Jκ-proximal (bottom) to Jκ-distal (top) genomic location. **(C)** Vκ gene rearrangement frequency arranged from Jκ-proximal (bottom) to Jκ-distal (top) with every other bar labeled on the left y-axis. Right side bars display VκJκALL gene frequencies, left side bars display VκJκ1 gene frequencies. Black bars depict deletional rearrangements, red bars depict inversional rearrangements. Genes with 0% rearrangement frequency in VκJκALL are excluded. Dotted vertical lines mark 1, 2, and 5% rearrangement frequency. **(D)** Rearrangement frequency ratios of VκJκ1/VκJκALL (left side) and the reciprocal VκJκALL/VκJκ1 ratios (right side). Vκ gene names listed are those that are >10-fold higher in VκJκALL vs. VκJκ1. All genes which had a VκJκALL frequency ≥0.05% in all 3 replicates are included. Vκ4-63Jκ1 was assigned a frequency equivalent to 1 read in order to calculate a ratio. **(E)** Jκ gene percent usage arranged in descending order of rearrangement frequency. The most highly rearranged genes are on top and the least frequently rearranged genes are at the bottom. Color coding as described in **(B)**. For **(A–E)**, data is derived from 3 independent pre-B cell gDNA biological replicates. For **(B,E)**, only Vκ genes which had at least 5 reads in each of the 3 gDNA biological replicates are included. SEM error bars plotted for **(C,D)**.

We observed that individual Vκ genes had very different Jκ usage, as observed before (5, 8), so we separated the gDNA repertoire data into the four groups (Jκ1, Jκ2, Jκ4, and Jκ5) (Figure 1A). The Jκ1 repertoire was the most divergent from the other Jκ repertoires whereas Jκ4 and Jκ5 were 97% identical. Jκ2 displayed higher similarity to Jκ4 and Jκ5 (91–94%) than to Jκ1. Figure 1B shows that biased Jκ usage occurs throughout the Igκ locus. Many genes displayed preferential Jκ1 gene usage, with the extreme being Vκ15-102 at 100%. Conversely, the frequently rearranging gene Vκ17-121 gene only rearranged to Jκ1 3% of the time. Jκ1 rearrangements are considered to represent the first rearrangements in most cases although primary rearrangements can probably be made to downstream Jκ genes (29). This is supported by the finding that RAG-mediated breaks at Jκ1 are observed at earlier times in pre-B cell differentiation than at Jκ4 and Jκ5 RSS sites, and thus Jκ4 and Jκ5 are usually associated with secondary rearrangements either in the case of a non-productive primary rearrangement or due to an autoreactive B cell receptor (29, 30). Examination of the Jκ1 repertoire (hereafter referred to as VκJκ1) allows investigation of most of the initial kappa rearrangements with the caveat that some Jκ1 rearrangements may have arisen on the second allele. Over half of the Vκ genes are in the opposite orientation from the Jκ-Cκ gene cluster. This means that some Igκ rearrangements will result in deletion of the intervening DNA while other rearrangements lead to inversion and thus retention of intervening Vκs for possible future use (31). Intervening gDNA from deletional rearrangements is retained in the cell as circular DNA and if created in pre-B cells, is PCR amplifiable since pre-B cells do not proliferate during rearrangement (32, 33). Secondary inversional rearrangements also retain previous VκJκ rearrangements in the Igκ locus itself. Primary Jκ1 rearrangements would be lost if a cell dies after multiple unsuccessful rearrangements, if excision circle DNA created in pro-B cells was diluted through pre-BCR-mediated proliferation, or if a productive and functional BCR rapidly entered the immature B cell compartment. Since we observe that 8 genes rearrange to Jκ1 0–3% of the time but rearrange to other Jκ genes in both pro-B and pre-B cells, this indicates that a few Vκ genes most likely make initial rearrangements to downstream Jκ genes (Dataset S2). Thus, examination of all Vκ gene usage (hereafter referred to as VκJκALL) as we do here allows one to examine overall rearrangement frequencies much more accurately.

One potential caveat of using VκJκALL is that different Jκ gene repertoires could be disproportionately represented based on possible Jκ primer biases that exist in the VDJ-seq protocol. However, we think our VκJκALL data lacks primer bias and is thus representative of the actual total rearrangements because different sets of Jκ PCR primers that we used yielded similar Jκ percentages, with Jκ1 accounting for over ~40% of gDNA rearrangements while the other Jκ genes contributed ~20% each (Figures S2D, S3B). We cannot exclude the possibility that a Jκ bias may have been introduced with the common set of Jκ biotinylated primers that we used. However, the recent VJ repertoire study using VDJ-seq employed different biotinylated and PCR primers, and still revealed a similar breakdown in Jκ usage (>35% Jκ1) albeit with slightly more Jκ2 representation relative to our data (8).

The repertoire data obtained from these gDNA libraries reveal highly uneven Vκ gene usage when examining individual Jκ gene repertoires, VκJκALL repertoires as well as within respective Vκ gene families (Figure 1C, Figures S4A,B). Genes that are underrepresented in VκJκ1 relative to VκJκALL are shown in Figure 1D. Uneven Jκ usage does not show any bias for Vκ genes that rearrange frequently or infrequently (Figure 1E). Jκ1 rearrangements were also more biased to deletional rearrangements than rearrangements using the other Jκ genes (Figure S3C).

We compared VκJκALL frequencies to the RSS quality score (RIC score). The RIC score is derived from an algorithm that predicts RSS site sequence quality based on the heptamer, spacer and nonamer (34). Consistent with prior studies of heavy chain and VκJκ1 light chain repertoire studies, our data also suggests the RIC score is the most important individual factor in predicting whether a V gene is active or inactive as determined by RF classification analysis (6–8). This is reasonable since a V gene cannot rearrange without a reasonable RSS. However, when only Vκ genes whose rearrangement accounts for a minimum of 0.01% of the repertoire were analyzed, linear regression analysis shows only a modest correlation (*R* = 0.33–0.41) between the RIC score and rearrangement frequencies (Figures S4C,D).

### Comparison of repertoires obtained from paired sets of gDNA and RNA

**Figure 2:**
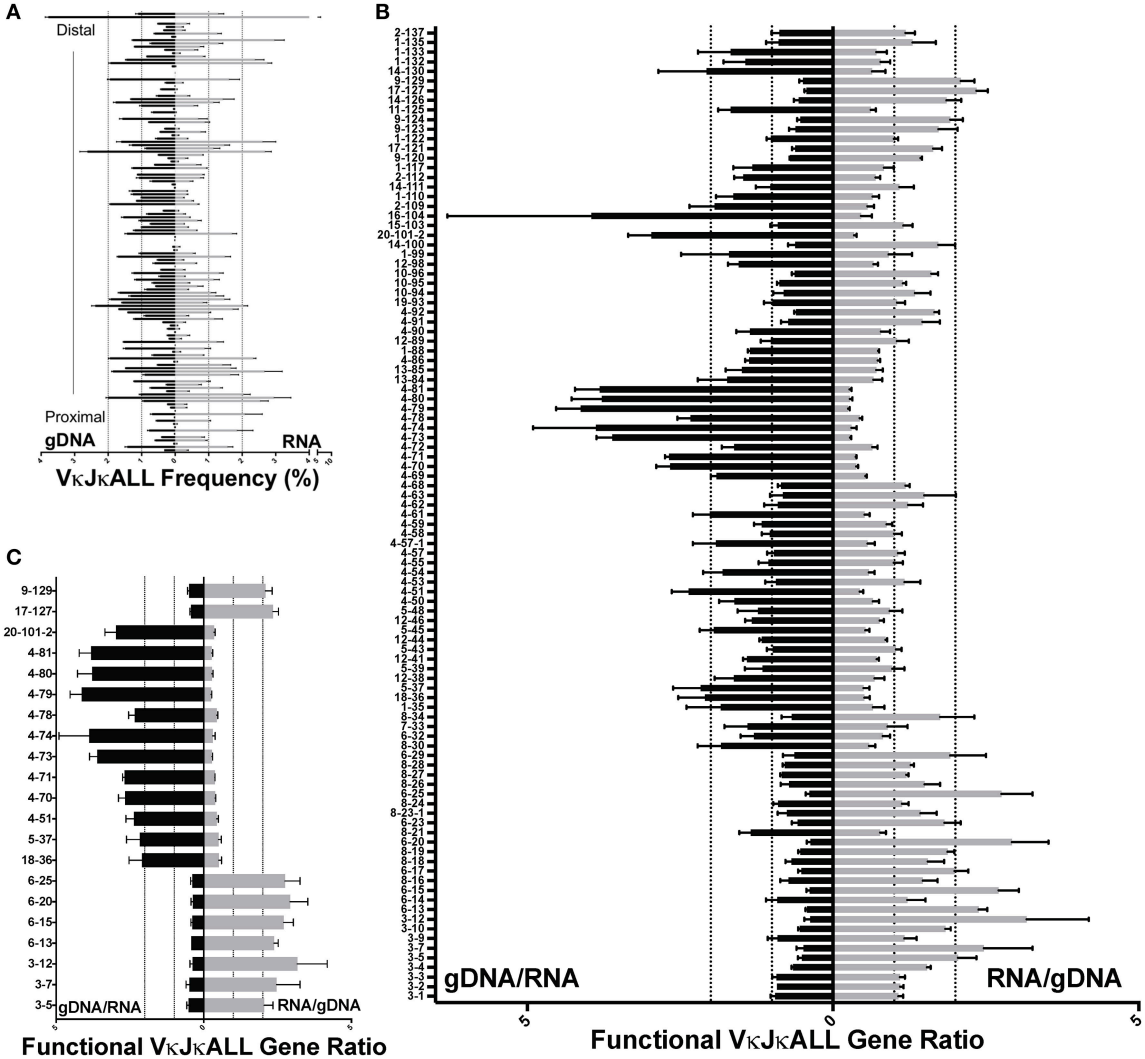
Comparison of VκJκALL gDNA and RNA repertoires in pre-B cells. **(A)** VκJκALL gDNA repertoire (left side) vs. VκJκALL RNA repertoire (right side) plotted in descending order from Jκ-distal (top) to Jκ-proximal (bottom) genomic location. Dotted vertical lines depict 1 and 2% frequencies. Genes with no reads in both gDNA and RNA repertoires are excluded. **(B)** Ratio of gDNA/RNA (left side) or RNA/gDNA (right side) for all IMGT-designated functional and ORF Vκ genes are arranged vertically as in **(A)**. Dotted vertical lines depict 1 (no difference) and 2-fold changes for both ratios. Only genes that had reads in all 3 biological replicates for both gDNA and RNA are included. **(C)** Functional VκJκALL genes that are ≥2-fold greater in gDNA (left) or ≥2-fold greater in RNA (right) repertoires and ≥1.5-fold greater in each individual comparison. For **(B,C)**, each pairing of gDNA and RNA derives from the same sorted pre-B cells. For **(A–C)**, 3 biological replicates are used in both gDNA and RNA data. Errors bars represent SEM.

In order to uncover any biases that might be present when comparing repertoire data from gDNA vs. RNA, we compared gDNA libraries described above to RNA repertoire libraries made from the same batches of sorted pre-B cells (Figure S1). The 3 libraries made from RNA were ~88% similar (Figures S2B,E). We directly compared RNA and gDNA from the same sorted cells. We first compared the frequency of usage of each Vκ gene in libraries made with gDNA and RNA (Figure 2A) using VκJκALL sequences to assess the total repertoire, although data using VκJκ1 was similar (Figure S5). The rearrangement frequencies derived from pre-B cell gDNA and RNA are unequal. To more effectively visualize potential biases, we calculated the ratios between the two for functional Vκ genes that had reads in both repertoires. These ratios reveal that ~80% of functional Vκ genes had RNA/gDNA or gDNA/RNA ratios that were on average no more than 2-fold higher for either gDNA or RNA repertoires (Figure 2B, Figure S5C). This suggests that these Vκ gene promoters are of similar strength. Figure 2C depicts only the ~20% functional Vκ genes (21 genes, pseudogenes have been removed) with ≥2-fold ratio of gDNA/RNA frequency or vice versa, with all replicates having ≥1.5-fold difference. We observe ≥2-fold RNA/gDNA frequency ratios (i.e., higher representation in RNA repertoire) for many proximal Vκ3 and Vκ6 family member genes and ≥2-fold gDNA/RNA frequency ratios (i.e., higher in the gDNA repertoires) for several Vκ4 family genes as well as 3 genes from other Vκ families; Vκ18-36, Vκ5-37, and Vκ20-101-2. We also observed a few Jκ-distal Vκ genes displaying an increased RNA/gDNA frequency ratio: Vκ17-127 and Vκ9-129. In summary, many proximal Vκ3 and Vκ6 family members plus few distal Vκ genes are overrepresented in the RNA-based libraries, while many central Vκ4 family members are underrepresented (Figures S6A,B). We hypothesize that many Vκ4 family members harbor weak promoters whereas many Vκ3 and Vκ6 family members have relatively strong promoters.

We observed the greatest disparity between gDNA and RNA Vκ gene rearrangements among IMGT classified pseudogenes, as would be expected (Figures S6C,D) since transcripts from pseudogenes with premature stop codons are subjected to increased RNA surveillance mechanisms (10), and indeed many are very reduced in the RNA repertoires. However, a few pseudogenes are well transcribed and rearrange frequently, such as Vκ4-77, which despite having a stop codon, has a gDNA/RNA ratio of ~2.9 and represents 1.93% of the VκJκALL gDNA repertoire (Figure S6D). Thus, individual Vκ pseudogenes display varying degrees of representation in the RNA repertoire.

### Enhancer epigenetic markings predict rearrangement frequency

To be able to better predict which factors influence individual Vκ gene rearrangement frequencies, we analyzed 29 ChIP-seq features from our own data and from publically available datasets from both pro-B and pre-B cells for epigenetic marks, TFs, chromatin modifiers and RAG1 binding, as well as transcription from 5 RNA-seq from pro-B cells and pre-B cells, and also RIC scores. First, we quantified individual ChIP-seq and RNA-seq signal intensities in 4 windows around and including each Vκ gene (Figure S7A, Materials and Methods). We performed the analysis on the gDNA repertoire data since it lacks potential promoter biases.

**Figure 3:**
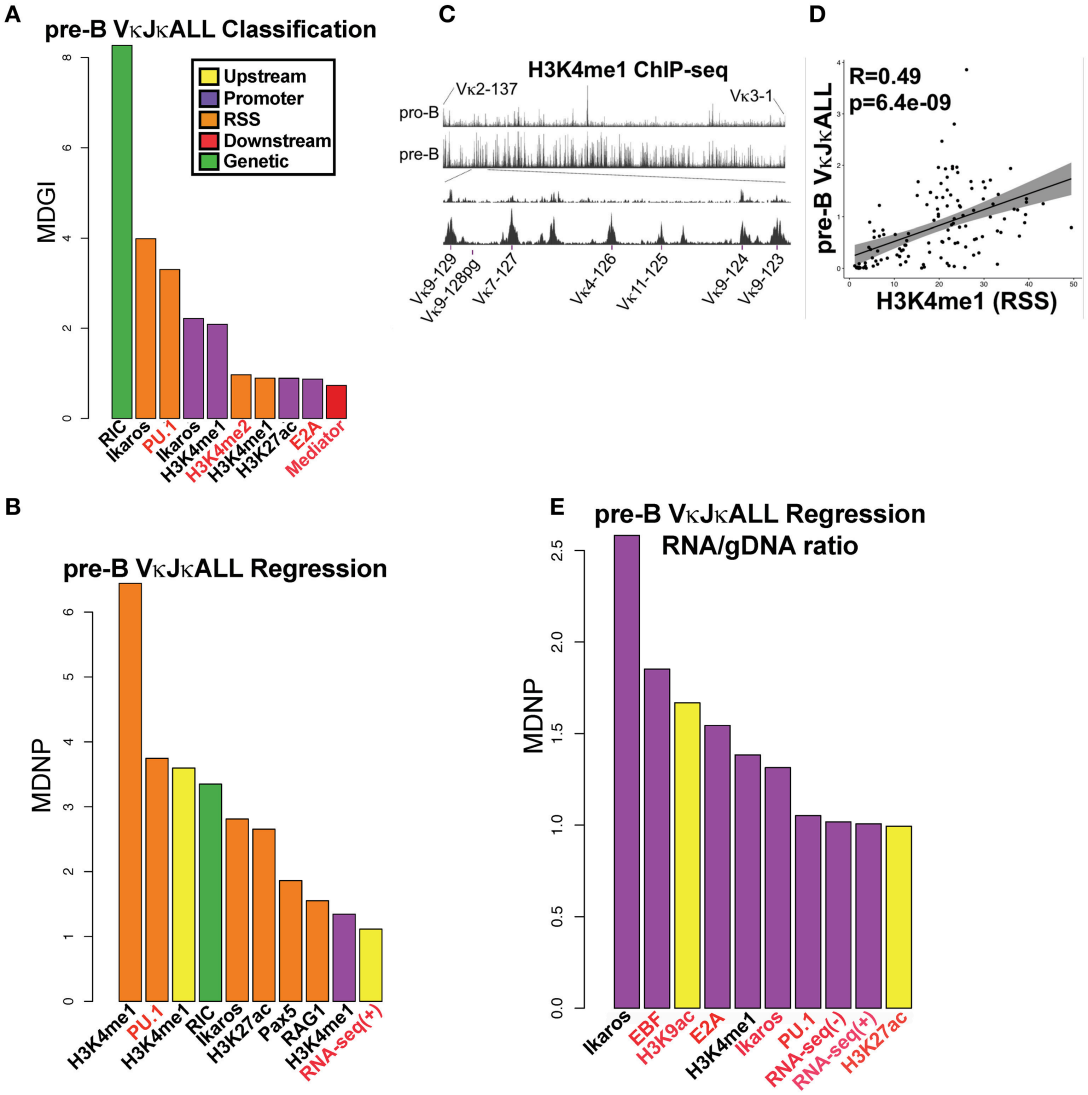
TFs predict rearranging Vκ genes and Vκ promoter strength while enhancer marks predict Vκ rearrangement frequency. **(A)** VI for each chromatin and RNA feature in a RF classification model for pre-B cell VκJκALL active/inactive genes. Vκ genes were categorized as active if they contained at least 15 reads per million reads among the VκJκALL read pool. Shown are the top ten features with significant VI. Mean Decrease in Gini Index (MDGI) is a measure of the decrease in accuracy if the feature is excluded. **(B)** VI for each chromatin and RNA feature in a RF Regression model for rearrangement frequency in pre-B cell VκJκALL active genes. Shown are features with significant VI. Mean Decrease in Node Purity (MDNP) is a measure of the decrease in accuracy if the feature is excluded. **(C)** H3K4me1 ChIP-seq data spanning the entire Vκ gene region comparing pro-B and pre-B cells (upper part). A zoom in of representative Jκ-distal region spanning genes Vκ9-123 to Vκ9-129 (lower part) illustrates the increase in H3K4me1 over Vκ genes in pre-B cells. Vκ9-128 is a pseudogene. **(D)** Scatterplot of H3K4me1 (within the RSS window) signal vs. VκJκALL pre-B gDNA rearrangement frequency, with linear regression line and 95% confidence interval using ggplot2 in R. Pearson correlation coefficient and associated p-value are also reported. **(E)** RF regression plot as in B for pre-B cell VκJκALL gene RNA/gDNA rearrangement frequency ratios. For **(A,B,E)**, chromatin or RNA feature is listed below bar. ChIP-seq/RNA-seq datasets derived from pro-B cells are denoted in red font. Black labeled features are derived from pre-B cells.

We first performed a classification analysis using Random Forest (RF) to examine which factors can predict whether a Vκ gene rearranges (active) or does not rearrange (inactive). We considered a Vκ gene active if it rearranged at least 15 times per million rearrangements within the gDNA VκJκALL repertoire. Our findings reveal that RIC score was most predictive of active vs. non-active genes, which is expected since genes with poor RSS are unlikely to rearrange efficiently. After RIC score, Ikaros binding within the RSS and promoter window and PU.1 binding in the RSS window were most predictive of the potential for active Vκ gene rearrangement (Figure 3A).

We next performed RF regression analysis to determine variable importance (VI) on only active pre-B cell genes within the gDNA VκJκALL repertoire to assess which factors influence individual Vκ rearrangement frequency levels. The epigenetic enhancer mark H3K4me1 in pre-B cells was most predictive of Vκ gene rearrangement levels, particularly at the 800 bp RSS window but also at upstream and promoter regions (Figure 3B). The levels of H3K4me1 are observed to dramatically increase at the pre-B cell stage over many Vκ genes. Figure 3C displays pro-B and pre-B cell H3K4me1 ChIP-seq data for the entire Igκ locus as well as a blow up of a representative Jκ-distal region. Linear regression analysis comparing H3K4me1 signal intensity within the RSS window and pre-B cell VκJκALL gDNA rearrangement frequency reveals a strong correlation with a Pearson correlation R value of 0.49 (Figure 3D). PU.1, considered a pioneering TF that initiates chromatin remodeling and subsequent H3K4me1 deposition (20), was the second highest predictor of rearrangement frequency. In addition, the active enhancer mark H3K27ac (35) and enhancer associated Ikaros and Pax5 scored high (36, 37). RAG1 binding at the RSS also scored high in the VκJκALL repertoire and was the highest predictive factor in influencing VκJκ1 rearrangement levels (Figure 3B, Figure S7B). Unlike the Igh locus where it was found that proximal V_H_ gene rearrangement levels correlated with proximity to architectural factors CTCF and the cohesin complex member Rad21 (6), our regression models do not indicate either as predictive of rearrangement levels. Further, minimum distance calculations of CTCF and Rad21 show that almost all Vκ genes are positioned at a significant distance from bound CTCF/Rad21 (Figure S7D). Overall, our data indicates that epigenetic marks of enhancers, especially H3K4me1 and TFs associated with enhancers, predict both active rearrangement status and influence individual Vκ gene rearrangement frequency.

We also performed regression analysis using pre-B cell RNA/gDNA ratios from VκJκALL rearrangement frequencies of IMGT classified functional Vκ genes to identify factors that may be responsible for elevated or decreased RNA representation relative to gDNA. Ikaros was the top factor, followed by EBF and E2A, all binding at the promoter window (Figure 3E). Promoter window signal intensity values for Ikaros, EBF, and E2A were 3.1- to 6.7-fold increased for the overexpressed Vκ3 and Vκ6 family genes vs. the underrepresented Vκ4 family genes. Values for all other Vκ families combined were intermediate for Ikaros and E2A binding, although EBF binding was similar for Vκ3/6 and the other non-Vκ4 families (data not shown). This data suggests binding of these 3 TFs at individual Vκ gene promoters is predictive of higher representation in RNA-based libraries relative to gDNA-based libraries.

In order to identify a minimum subset of features that together best predict active vs. inactive Vκ genes, active Vκ rearrangement frequencies or RNA overrepresentation, we performed a feature selection, using Recursive Feature Elimination (RFE) analysis, using all possible combinations of the 20 most important features from the initial RF classification and regression models. It is important to note that while this analysis does not necessarily mean that these features are the most important individually (compared to VI barplots), it suggests that together they are able to explain the largest proportion of the variability in the data. Ikaros and PU.1 binding at the RSS along with the RIC score were among the 4 factors that when used together are the most predictive for pre-B cell active vs. inactive Vκ genes (Figure S7E). For determining which factors together could best predict the level of rearrangement of individual active Vκ genes, H3K4me1 and PU.1 at the RSS were among the top three factors (Figure S7F). Lastly, Ikaros, EBF, and E2A at the promoter were among the top 6 factors that could most accurately predict high RNA/gDNA ratios (Figure S7G). Feature selection analysis has identified a few variables that when considered together are able to predict active Vκ rearrangement levels and promoter strengths with an accuracy comparable to the full model. The features identified are consistent with the VI data extracted from the initial RF models (Figures 3A,B,E).

### The earliest Igκ rearrangements made in pro-B cells are more Jκ-distal biased

**Figure 4:**
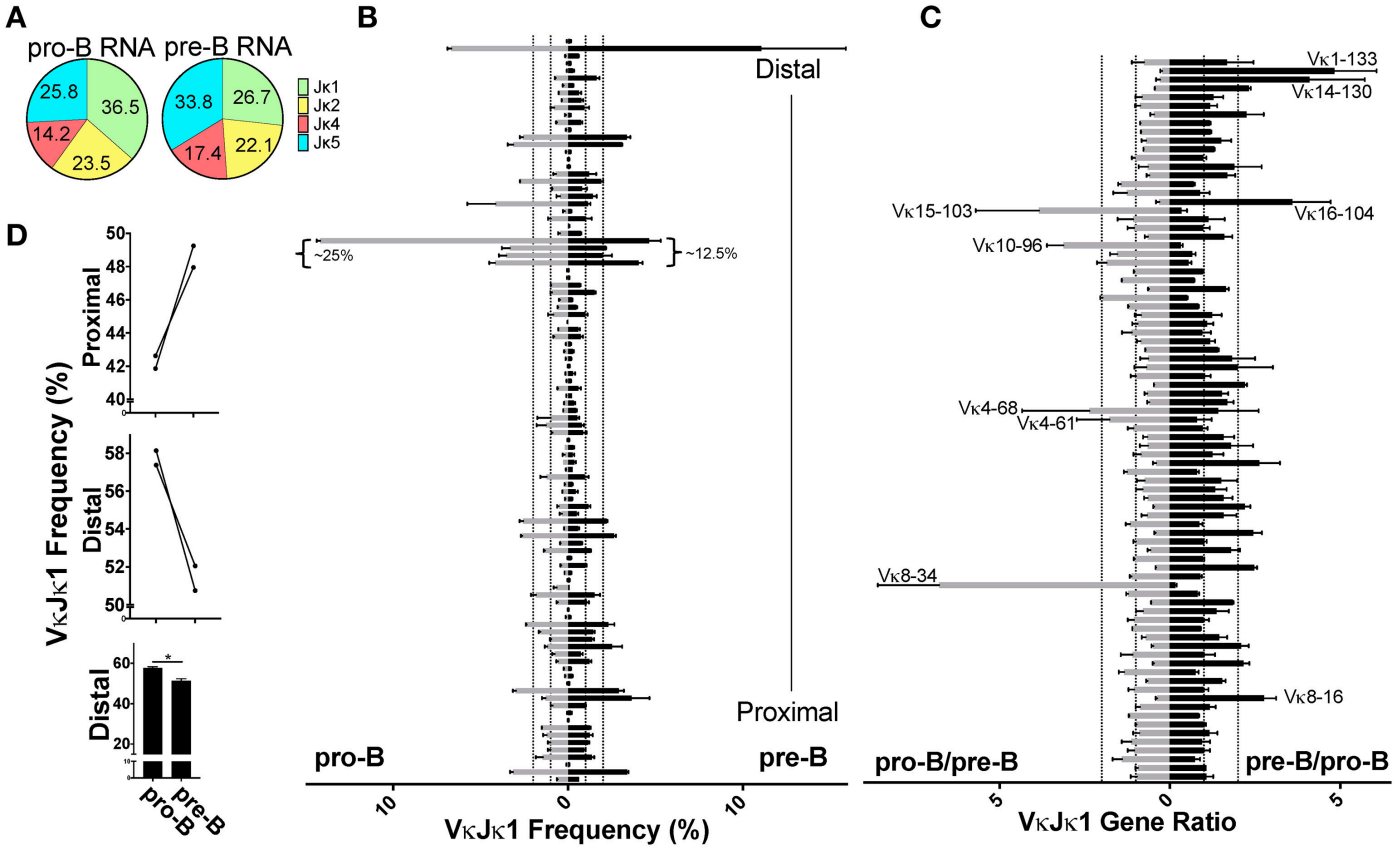
Pro-B cell VκJκ1 RNA repertoire is biased to usage of the Jκ-distal half of the locus. **(A)** Pie chart indicating average percent Jκ gene usage in pre-B (3 biological replicates) and pro-B (2 biological replicates) cells. **(B)** Relative rearrangement frequencies in the VκJκ1 RNA repertoires of pro-B (left) and pre-B cells (right) arranged from Jκ-distal (top) to Jκ-proximal (bottom). Dotted vertical lines represent 1 and 2% rearrangement frequencies. Brackets mark the frequently rearranging Vκ genes 19-93 to 10-96. Only Vκ genes with at least one read in each sample are shown. **(C)** VκJκ1 rearrangement frequency ratios of pro-B/pre-B (left) and the reciprocal pre-B/pro-B (right) arranged vertically as in **(B)**. Dotted vertical lines represent 1 (no difference) and 2-fold changes in rearrangement frequency. Only Vκ genes where both pro-B cell replicates had 0.05% frequency or greater are shown. For **(B,C)**, only the 2 pre-B cell replicates that can be paired (from same sort) with the 2 pro-B cell replicates are used. **(D)** Comparison of pro-B and pre-B cell VκJκ1 RNA rearrangement frequency in the Jκ-distal half vs. Jκ-proximal half of the Igκ locus. Jκ-proximal Vκ genes include genes Vκ3-1 to Vκ13-76 (upper bar graph). Jκ-distal Vκ genes include Vκ4-77 to Vκ2-137 (center bar graph). Each line connects pro-B and pre-B cell values from the same mice. Lower bar graph compares the combined distal frequencies for both pro-B or pre-B cell replicates. Error bars represent SEM. *p < 0.05.

Although most Igκ rearrangements occur at the pre-B cell stage, an estimated 15% of CD43^+^ pro-B cells undergo early Igκ rearrangement (13). To examine whether early first pro-B Igκ rearrangements differed from that of the full Igκ repertoire generated in pre-B cells, we twice sorted both cell types from the same pool of mice and prepared gDNA and RNA libraries from each (Figure S1A). We first analyzed pro-B cell RNA repertoires (Figure S2F) which had many more reads than pro-B cell gDNA libraries. Pro-B cells had increased Jκ1 usage relative to pre-B cells, so we focused our analysis on the VκJκ1 repertoire (Figure 4A, Figure S3B) although similar trends were observed in VκJκALL. Vκ10-96 was the most frequently rearranging gene in the pro-B cell RNA repertoire, roughly 3 times higher than its representation in the pre-B cell RNA repertoire (Figure 4B, Figure S8A). In fact, the 4 Vκ genes from 19–93 through to 10–96 represented ~25% of the entire pro-B cell VκJκ1 RNA repertoire vs. ~12.5% in pre-B cells.

Comparing the pro-B/pre-B Vκ RNA ratios, we observe about 17 Vκ genes that are ≥2-fold in rearrangement frequencies between pro-B and pre-B cells (Figure 4C, Figure S8B). To examine if there was any general bias between the pro-B/pre-B RNA ratios, we categorized Vκ RNA frequency as belonging to the Jκ-proximal half or Jκ-distal half of the Igκ locus using the genomic distance between the most Jκ-proximal Vκ gene (Vκ3-1) to the most Jκ-distal Vκ gene (Vκ2-137) to calculate the division between the proximal and distal halves of the locus. Vκ3-1 through Vκ13-76 were considered proximal half Vκ genes and Vκ4-77 through Vκ2-137 were distal half Vκ genes. Pro-B cells displayed roughly 5% greater overall frequency in rearrangements to the distal half of the Igκ locus relative to pre-B cells (Figure 4D, Figure S8C). Thus, pro-B cell RNA repertoire reflects skewing to the distal half of the Igκ locus, largely due to the contribution of Vκ10-96.

**Figure 5:**
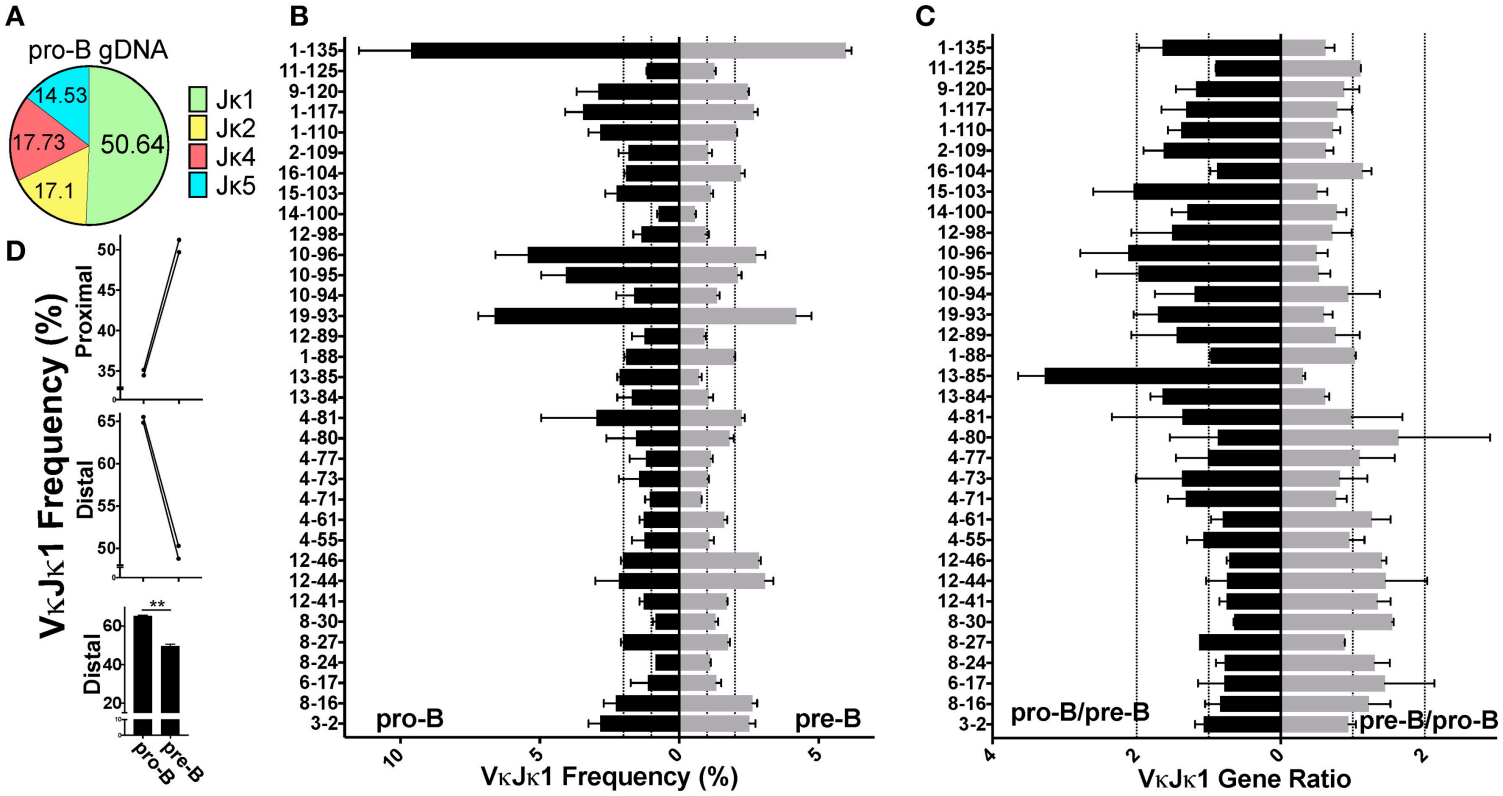
Pro-B cell gDNA rearrangements are biased to the Jκ-distal half of the kappa locus. **(A)** Pie chart indicating average percent Jκ gene usage in pro-B cells, from 2 biological replicates. **(B)** VκJκ1 gDNA repertoire frequencies of pro-B (left) and pre-B cells (right) arranged from Jκ-distal (top) to Jκ-proximal (bottom). Dotted vertical lines represent 1 and 2% rearrangement frequencies. Only Vκ genes comprising at least 0.5% of total rearrangements in both pro-B replicates were included. **(C)** VκJκ1 gDNA ratios of pro-B/pre-B (left) and the reciprocal pre-B/pro-B ratio (right) arranged vertically as in **(B)**. Dotted vertical lines represent 1 (no difference) and 2-fold changes in relative gDNA rearrangement. **(D)** Comparison of pro-B and pre-B cell VκJκ1 rearrangements in the Jκ-distal half vs. Jκ-proximal half of the kappa locus as in 4D. Error bars represent SEM. **p < 0.01.

Despite low read numbers from pro-B cell gDNA libraries, enough reads were obtained to assess actual rearrangement differences between pro-B and pre-B cells, since the gDNA repertoire is the most unbiased for the reasons stated above. We observed that pro-B cells are more skewed toward deletional rearrangements than pre-B cells which is in agreement with their more predominant use of distal Vκ genes, most of which are in the deletional orientation (Figure S3C). Pro-B cells use Jκ1 in 50% of rearrangements compared with 40% for pre-B cells (Figure 5A, Figure S3B), similar to the Jκ1 skewing observed in the pro-B cell RNA repertoire. Because of this Jκ1 bias, we focused on VκJκ1 rearrangements, but data was similar with VκJκALL analysis. Similar to the RNA repertoire analysis, Vκ19-93 through Vκ10-96 accounted for a large part of the pro-B cell VκJκ1 gDNA repertoire (~17.5%). However, Vκ1-135 rearrangements are more predominant in pro-B cell gDNA and accounted for ~10%of VκJκ1 rearrangements (Figure 5B, Figure S8D), representing a 1.6-fold increase compared to pre-B cells. gDNA rearrangement frequency ratios indicate Igκ distal half Vκ rearrangements are much more pronounced in pro-B cells (Figure 5C, Figure S8E), up 15% compared to pre-B cells (Figure 5D, Figure S8F). Thus, pro-B cell rearrangements are biased to the distal half of the Igκ locus.

We also compared matched pro-B cell gDNA and RNA repertoires and found that similar to pre-B cells, many Vκ4 family gene members were underrepresented and Vκ6-15 was overrepresented in the RNA repertoire (many Vκ3/6 family genes were filtered out due to low read numbers) (Figures S9A,D). Ratio analysis reveals similar trends as observed in pre-B cells (Figures S9B,C, E,F). Thus, promoter strength differences appear to be consistent through early B cell development.

### PU.1 binding at the promoter predicts pro-B cell Vκ relative rearrangement frequencies

**Figure 6:**
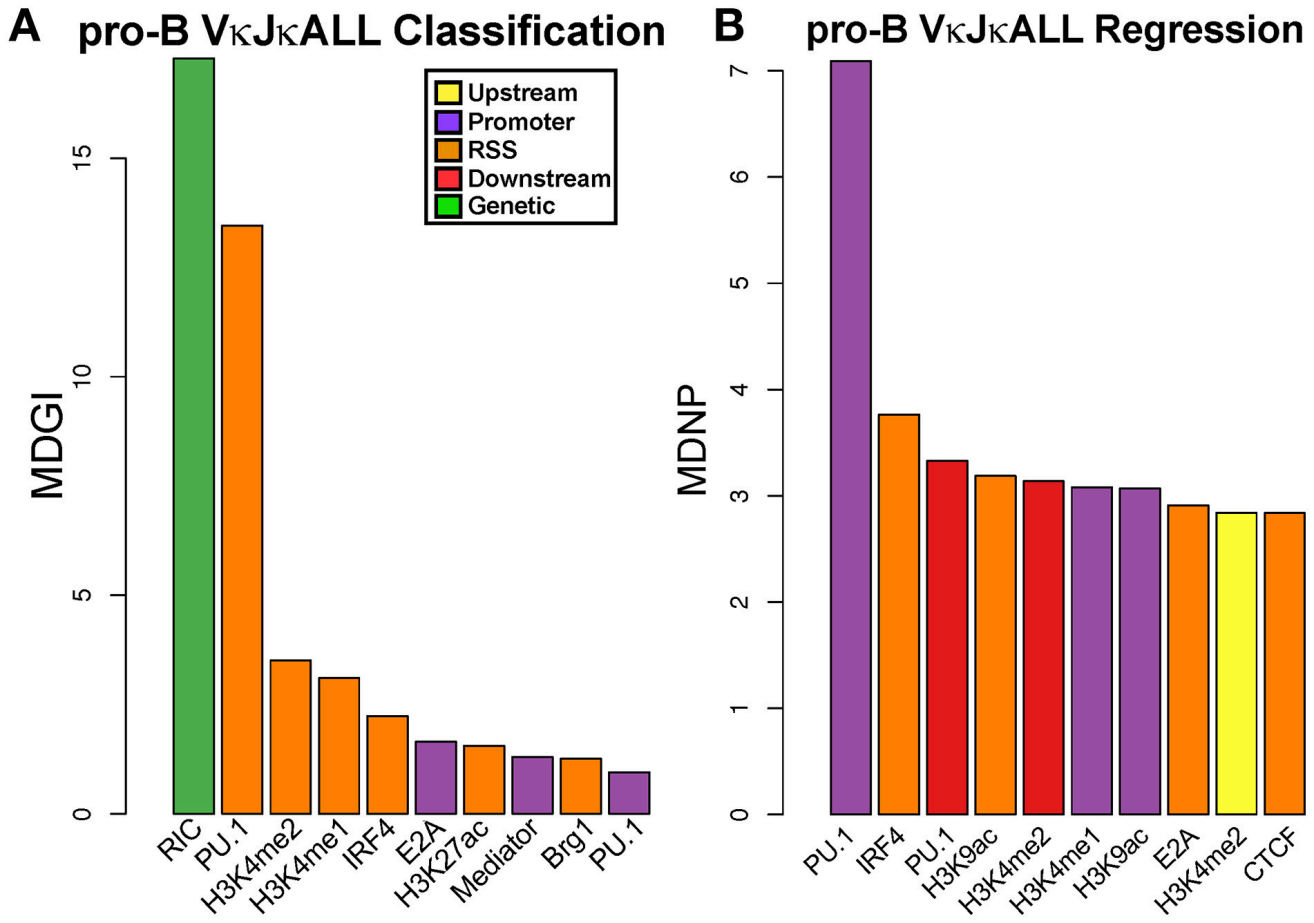
PU.1 predicts active Vκ genes and rearrangement frequency in pro-B cells. **(A)** VI for each chromatin and RNA feature in a RF classification model for pro-B cell gDNA VκJκALL active/inactive genes. Vκ genes were categorized as active if they contained at least 1 read. Shown are the top ten features with significant VI. **(B)** VI for each chromatin and RNA feature in a RF regression model for rearrangement frequency in pro-B cell gDNA VκJκALL active genes. Shown are features with significant VI. For **(A,B)**, chromatin or RNA feature is listed below bar. All features used in RF are from pro-B cells.

We performed the same RF analysis as with pre-B cells. Classification analysis on pro-B cell gDNA repertoire data revealed that just as in pre-B cells, RIC score followed by PU.1 binding in the RSS window was most predictive of active vs. non-active Vκ genes (Figure 6A). However, unlike pre-B cells, regression analysis shows PU.1 binding at the promoter is by far the biggest predictor of rearrangement levels among active Vκ genes in pro-B cells (Figure 6B, Figure S7C). Also, enhancer marks are not as predictive of pro-B cell Vκ rearrangement frequency although H3K4me2 followed PU.1 in relative importance. Feature selection analysis was performed as with the pre-B cell data set. PU.1 at the RSS window was among the top features that when considered together were most able to accurately predict both active Vκ genes (vs. inactive Vκ genes) and also Vκ rearrangement frequency levels (Figures S7H,I), consistent with our VI analysis (Figure 6). Overall, 3–9 factors together could reasonably predict pro-B cell active Vκ genes (vs. inactive Vκ genes) and active Vκ rearrangement frequency levels, respectively.

### iEκ regulates individual Vκ gene usage

**Figure 7:**
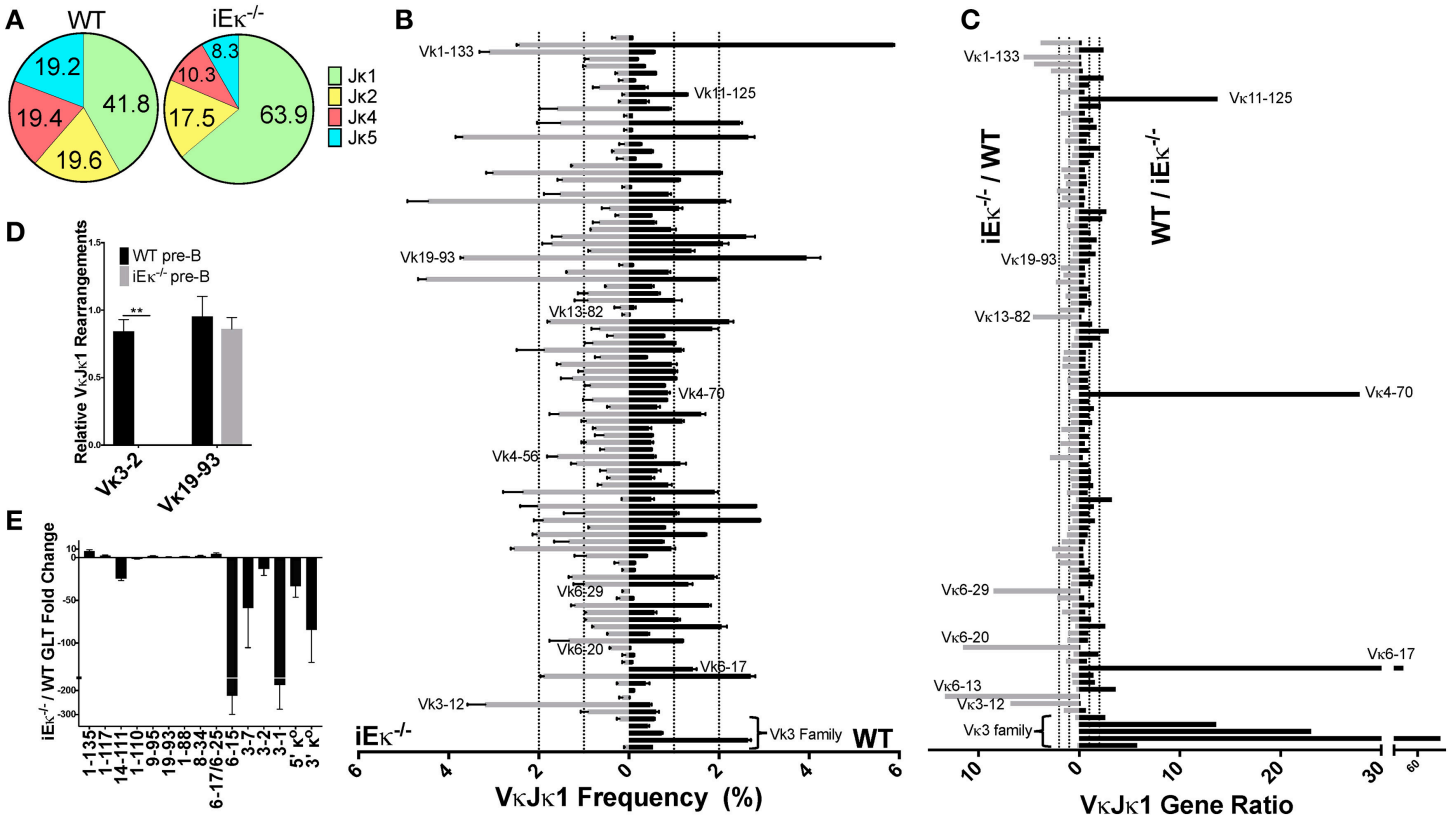
iEκ regulates rearrangement of Vκ3 family genes. **(A)** Pie chart indicating the percent Jκ gene usage in WT and iEκ^−/−^ pre-B cells. Data for WT is a combination of all 3 biological replicates, while the data for iEκ^−/−^ represents a combination of 2 replicates. Percent Jκ usage is indicated. **(B)** VκJκ1 gene gDNA rearrangement frequency in WT (right) and iEκ^−/−^ (left) pre-B cells. Labeled Vκ genes were most affected by the absence of iEκ. Only Vκ genes that are present at 0.1% or more in either WT or iEκ^−/−^ cells are listed. Data are comprised of 2 WT replicates and 2 iEκ^−/−^ replicates. **(C)** VκJκ1 gDNA gene ratios comparing WT and iEκ^−/−^ cells, iEκ^−/−^/WT on the left, WT/iEκ^−/−^ on the right. The 2 replicates of WT or iEκ^−/−^ were combined to produce one value for a Vκ gene ratio. Only VκJκ1 genes that are at least 0.1% in WT are listed. Vκ genes in iEκ^−/−^ pre-B cells which had a rearrangement frequency of 0 (when paired with WT genes that had a frequency ≥ 0.1) were replaced with the frequency equivalent of 1 read. Therefore, some of the WT/iEκ^−/−^ ratios are an underrepresentation of the actual difference between the two. **(D)** TaqMan qPCR on sorted pre-B cell WT and iEκ^−/−^ gDNA for Vκ 3-2Jκ1 or Vκ19-93Jκ1. Data was first normalized to heavy chain Eμ and then normalized to the total rearrangements using a degenerate Vκ binding primer “VκALL” (Materials and Methods). **p < 0.01. (E) qPCR showing GLT expression throughout the Igκ locus in CD19^+^ purified BM pre-B cells from iEκ^−/−^ Rag^−/−^ hIgH Tg and Rag^−/−^ hIgH Tg control mice. Bars below the x axis indicate lower expression in iEκ^−/−^ Rag^−/−^ hIgH Tg relative to Rag^−/−^ hIgH Tg control pre-B cells. Bars above the x axis indicate higher expression in iEκ^−/−^ Rag^−/−^ hIgH Tg pre-B cells. GLT expression was normalized to GAPDH. GLT data covering Vκ3-7, Vκ1-110, Vκ1-111, and Vκ1-117 genes was derived from 2 biological replicates, all other GLT data was derived from 3 biological replicates. **(B–E)** Error bars represent SEM.

iEκ is very important, but not absolutely required for Igκ chain rearrangement (15). However, whether iEκ can influence individual Vκ gene usage has not been examined previously. We therefore performed gDNA repertoire analysis from sorted pre-B cells from iEκ^−/−^ mice from 2 biological replicates, which were 87% similar (Figure S2G). We observed that iEκ^−/−^ pre-B cells had a dramatic increase in the relative frequency of Jκ1 gene usage, at over 63% compared to 41% for WT pre-B cells (Figure 7A, Figure S3B). For this reason, we focused our analysis on the VκJκ1 repertoire but VκJκALL data was similar. Strikingly, we find iEκ^−/−^ pre-B cells have a dramatically reduced representation of Vκ3 family proximal genes (Vκ3-1 to Vκ3-9) relative to WT (Figure 7B, Figure S10A). At Vκ3-10, both pre-B cell strains approach parity. At Vκ3-12, iEκ^−/−^ pre-B cells are >3-fold higher in rearrangement than WT cells. Other genes scattered throughout the locus were also altered. Vκ genes 6-20, 6-29, 13-82, and 1-133 represented a higher percentage of the repertoire in iEκ^−/−^ pre-B cells. Conversely, Vκ genes 6-17, 4-70, and 11-125 were diminished in iEκ^−/−^ frequency. These differences are more easily observed when displaying the ratio of WT to iEκ^−/−^ frequencies or vice versa (Figure 7C, Figure S10B). This data provides evidence that iEκ is able to regulate not only overall levels of rearrangement but is able to regulate individual Vκ usage.

To confirm the dramatic discrepancy in Jκ-proximal Vκ3 family gene rearrangements between WT and iEκ^−/−^ pre-B cells, we assayed sorted pre-B cell gDNA using TaqMan qPCR with a Jκ1 reverse primer and Jκ1 probe. In addition to normalizing data for DNA loading (Eμ), the data were also normalized to the total level of rearranged Vκ genes (using a degenerate Vκ primer) since overall levels of kappa rearrangement are far lower in iEκ^−/−^ pre-B cells relative to WT pre-B cells (15). We quantified a 6.5-fold decrease in iEκ^−/−^ pre-B cell rearrangement relative to WT pre-B cells using the degenerate Vκ primer with the Jκ1 reverse primer and probe (data not shown). However, this is likely an underestimate since downstream Jκ usage in WT pre-B cells accounts for a bigger proportion of total rearrangements relative to iEκ^−/−^ pre-B cells. By normalizing Vκ gene usage to the total level of Vκ rearrangements, we are interrogating how the frequency of a given Vκ gene changes within the pool of rearrangements that do occur. We examined the Vκ3-2 gene due to it its elevated Jκ1 rearrangement frequency (~2.5%) in WT pre-B cells. In contrast to WT, no detectable Vκ3-2 rearrangement was found in iEκ^−/−^ pre-B cells (Figure 7D). Vκ19-93, a highly rearranged gene whose relative frequency is similar in WT and iEκ^−/−^ pre-B cells, is shown to be proportionately unchanged upon iEκ deletion. We conclude that iEκ directly regulates the rearrangement of the most Jκ-proximal Vκ3 family genes.

The accessibility hypothesis postulates that germline transcription (GLT) correlates with accessibility for rearrangement (38). We hypothesized that the dramatic decrease in Vκ3 family proximal rearrangements observed in iEκ^−/−^ pre-B cells might be linked to decreased local transcription. We examined the transcriptional influence of iEκ deletion using Rag1 deficient, heavy chain transgenic mice which resemble pre-B cells but contain a germline Igκ locus configuration (16). These mice were crossed with iEκ^−/−^ mice to assess Vκ GLT in the absence of this enhancer. As can be seen in Figure 7E, iEκ absence results in a dramatic loss of GLT in the most Jκ-proximal Vκ region relative to WT pre-B cells. This transcriptionally deficient region contains the Vκ3 family genes that did not rearrange in iEκ^−/−^ pre-B cells. Thus, iEκ controls rearrangement and GLT of the proximal Vκ genes.

## Discussion

Our study is the first to directly compare Vκ gene rearrangement frequencies from both gDNA and RNA from paired samples of pro-B and pre-B cells. Our data shows that ~20% of functionally classified Vκ genes have ≥2-fold apparent differences in the frequency of rearrangements when comparing data obtained from gDNA vs. RNA repertoires in pre-B cells. We also show that individual Vκ genes display extremely varied Jκ gene usage, consistent with previous data (8). Additionally, to our knowledge, our study is the first to perform deep sequencing of the Igκ repertoire in WT pro-B cells and in iEκ^−/−^ pre-B cells, where we show that iEκ is critical for the rearrangement of Jκ-proximal Vκ3 family genes and that pro-B cell rearrangements are more biased to the distal half of the Igκ locus.

A previous study of B220^+^ BM B cells (primarily assaying pre-B cells and immature B cells) using 5′ RACE PCR revealed 7 genes that were highly represented among all Vκ genes, each ranging from 5 to 7% of the total repertoire (5). Our pre-B cell RNA dataset detected 130 genes with at least one read with 11 genes appearing at a frequency of 2% to just under 7%. Six of the seven top genes from the previous study were among our highest frequency gene list. Comparison of the two studies reveals that Vκ genes 9-120, 19-93, 6-23, 6-17, and 6-15 all increased ≥2-fold in frequency in that study compared to our repertoire data, perhaps being upregulated in the differentiation step between pre-B and immature B-cells. The other recent Igκ repertoire study examined gDNA rearrangements at the pre-B cell stage and this study also found unequal Vκ and Jκ gene usage (8). However, that study only analyzed VκJκ1 repertoire, whereas we analyzed the entire repertoire since we found that some Vκ genes rarely if ever rearrange to Jκ1.

We reasoned that direct comparison of gDNA and RNA repertoires would reveal any biases that might arise from differential promoter strengths of individual Vκ genes. We show that 80% of functional pre-B Vκ gDNA/RNA or RNA/gDNA frequency ratios are within 2-fold of each other. This suggests that most Vκ gene promoters share similar strengths. However, several Vκ3/6 family gene members as well as 2 Jκ-distal genes Vκ17-127 and Vκ9-129 had ≥2-fold higher representation in RNA repertoire. Conversely, many Vκ4 family members as well as Vκ20-101-2 were ≥2-fold lower in their RNA repertoire. Using regression analysis, we show that Ikaros, EBF and E2A binding to the promoter region predict high RNA representation and are thus likely drivers of strong Vκ promoters. Also, Vκ4 family gene members display greater genomic distance between the octamer and the TATA box compared to other Vκ family members (11). Increasing genomic distance between the octamer and TATA box has been shown to reduce transcriptional output using β-globin constructs (39). V_H_ promoter strength and TF complex formation *in vitro* decreases when increasing the distance between the octamer and another promoter motif called the heptamer (40). The increased distance between octamer and TATA box may explain why Vκ4 family gene members have lower RNA levels per rearranged gene. However, despite the fact that Vκ gene family promoters have different apparent arrangements of cis-regulatory elements, most appear to have similar promoter strengths or are brought to the same transcriptional capacity by the 3′ enhancer (3′Eκ) and Ed, which have been shown to be the primary regulators of the level of rearranged VκJκ transcription for most Vκ genes (14, 41–43). Relatively equal transcription of rearranged Vκ genes has implications for central tolerance since altered BCR levels or BCR signal intensity can profoundly impact central tolerance (44, 45).

We used machine learning RF analysis combined with a much larger ChIP-seq and RNA-seq database than any previous studies to reveal factors predictive of active vs. inactive Vκ genes (RF classification) and also individual Vκ gene rearrangement frequencies (RF regression analysis). Datasets were derived from both pre-B cells and pro-B cells since Igκ locus contraction, and to a minor extent rearrangement, occur at the pro-B cell stage (13, 46, 47). After RIC score, PU.1 binding (RSS window) and Ikaros (RSS and promoter windows) were identified as the highest predictors of active vs. inactive Vκ genes. Although PU.1 RSS binding was also identified as a high mark in a previous Igκ RF classification analysis (8), that study did not examine pre-B cell Ikaros binding. Regression analysis showed that the levels of the enhancer mark H3K4me1 in pre-B cells were most predictive of active Vκ gene rearrangement levels while RAG-1 binding within the RSS window predicted VκJκ1 pre-B cell rearrangement levels. This is the first report to show that H3K4me1 in pre-B cells, and active enhancer mark H3K27ac to a lesser extent, is the most predictive of individual Vκ gene rearrangement levels in pre-B cells. We also show that the extent of H3K4me1 greatly increases in pre-B cells compared to pro-B cells, particularly near Vκ genes. The presence of H3K4me1 levels at enhancers has been shown to correlate with long-range chromatin contacts (12). Thus, the degree of H3K4me1 at individual Vκ genes may facilitate long-range interactions responsible for locus contraction, and for rearrangement of that particular Vκ gene, providing a mechanistic explanation for higher rearrangement frequencies.

This is also the first report, to our knowledge, to identify PU.1 as a crucial regulator of Igκ rearrangement at the pro-B cell stage. This is consistent with a recent report showing that PU.1 regulates Igκ transcription and rearrangement in a pro-B cell line mainly by binding in close proximity to Vκ gene transcriptional start sites (48). Previous reports comparing pro-B cell ChIP-seq data sets with Igh gene rearrangement frequencies highlight that Vκ rearrangements are regulated in a distinct manner from V_H_ rearrangements. We previously demonstrated that proximity of CTCF and Rad21 was critical for proximal V_H_ gene rearrangements, while distal V_H_ gene rearrangement levels were predicted by high active histone marks (especially H3K4me2/3) (6). A more recent report identified Pax5 and IRF4 binding at the RSS as predictive of distal V_H_ gene rearrangement frequency (7). Unlike the Igh locus, neither CTCF or Rad21 binding appear to correlate with individual Vκ gene rearrangement frequency which is not unexpected since CTCF bound sites are not close to Vκ genes (Figure S7D) (49). Overall, our data reveal different mechanisms controlling rearrangement at the Igh vs. the Igκ locus as well as differential control of Vκ gene rearrangement in the pro-B cell stage vs. pre-B cell stage.

We observed higher Jκ1 usage in pro-B cells which we hypothesized was a result of these cells not having had as much time as pre-B cells to undergo additional rearrangements to downstream Jκ genes. We also observed that pro-B cells have a distinct bias for rearrangement to Vκ genes in the distal half of the kappa locus largely due to Vκ10-96 and Vκ1-135. A potential reason for this bias is that there is a higher proportion of long-range interactions between the Jκ/iEκ region and the Jκ-distal half of the Igκ locus vs. the Jκ-proximal half in pro-B cells, as assayed by 4C (E.M. Barajas-Mora, EK, AJF, manuscript submitted). This hypothesis would be consistent with the link between long-range interactions and rearrangement frequency (46).

Many long-range chromatin interactions are primarily mediated through CTCF (50, 51). CTCF binds to two cis-regulatory elements in the VJ intervening sequence that play important roles in Igκ rearrangement. These two elements, Cer (contracting element for recombination) and Sis (silencer in the intervening sequence), have overlapping but distinct functions at the kappa locus (52–55). Deletion of each element separately reveals that they mediate Jκ-distal Vκ rearrangement. However, only Cer is responsible for regulating locus contraction and its absence has a much more profound effect on repertoire composition. In addition to these two elements, CTCF binds to ~65 CTCF binding sites throughout the Vκ portion of the Igκ locus in pre-B cells. However, CTCF binding to the Igκ locus at the pro-B cell stage is much more restricted occurring mostly in the Jκ-distal half of the locus (49, 56), possibly partially explaining the preponderance of long-range interactions to the distal half of the locus in pro-B cells.

The highest observed pro-B cell CTCF and cohesin ChIP-seq peak occurs between the Vκ10-95 and Vκ10-96 genes. CTCF-mediated looping occurs predominantly when two CTCF sites are in convergent orientation (facing each other) as opposed to tandem orientation (both facing the same direction) (57, 58). The two CTCF sites in the Cer element both are oriented toward the Vκ genes, while the CTCF peak downstream of Vκ10-96 faces toward Cer. Preliminary 4C data from the viewpoint of this CTCF site shows a prominent interaction with the Cer element at the pro-B cell stage (E.M. Barajas-Mora, EK, AFJ, unpublished data). Because Cer regulates Jκ-distal Vκ gene usage (52), we hypothesize that a major contributing factor to elevated Vκ10 family member gene rearrangements in pro-B cells, especially Vκ10-96 but also Vκ19-93, Vκ10-94, and Vκ10-95 is the long-range interactions between this CTCF site and Cer that predominate over other Igκ locus interactions.

Another prominent pro-B cell Jκ-distal CTCF site is found near Vκ2-137 (49, 56). This CTCF is significant because it is relatively close (54 kb) to the Vκ1-135 gene which represents ~10% of all pro-B cell gDNA rearrangements. Even though individual Vκ gene proximity to CTCF does not predict rearrangement frequency, CTCF-mediated long-range interactions are likely in part responsible for the pro-B cell bias toward Jκ-distal Vκ genes, consistent with data showing that conditional early B cell deletion of CTCF leads to increased usage of proximal Vκ genes (59).

Lastly, we show that iEκ regulates usage of Vκ genes that lie within a region of iEκ-controlled GLT. The most Jκ-proximal Vκ genes within this transcriptionally deficient area in iEκ^−/−^ pre-B cells barely rearranged. However, we note that not all genes within this transcriptional sphere of iEκ influence are deficient in rearrangement. Genes at the Jκ-distal end of this enhancer-controlled transcriptional region did not display noticeable rearrangement defects (e.g., Vκ6-15) indicating a lack of strict correlation between GLT levels and rearrangement. Work from our lab using Cer-deleted Abelson-MuLV-transformed pro-B cell lines further indicates that the level of Vκ3 family gene rearrangement is not dependent on the level of GLT occurring over the gene body (60). Because strong iEκ to Vκ3 region interactions occur in pro-B cells (46) (E.M. Barajas-Mora, EK, AJF, manuscript submitted), a likely explanation of our data then is that the Vκ3 family genes that do not rearrange in the absence of iEκ are dependent on this enhancer for long-range contacts to drive rearrangement. However, compensatory long-range interactions in the absence of iEκ may occur and could explain altered rearrangement of other Vκ genes in both the proximal and distal half of the Igκ locus (e.g., Vκ6-29, Vκ4-70, Vκ11-125, and Vκ1-133). Both iEκ and the 3′Eκ enhancers have been shown to make long-range interactions throughout the Igκ locus in pre-B cells (46, 61). Additionally, both enhancers have partially redundant roles in kappa rearrangement, although iEκ is more important. Combined loss of both enhancers abrogates Igκ rearrangement entirely (14). If 3′Eκ were to exhibit altered bias in long-range interactions relative to iEκ, then Vκ gene rearrangement might be altered in the absence of iEκ. Another notable observation in the iEκ^−/−^ pre-B cells was the sizeable increase in Jκ1 usage compared to wild-type, ~63 vs. ~41%, respectively. Jκ1 rearrangements in iEκ^−/−^ are even more predominant than WT pro-B cells, in which Jκ1 represented 50% of all rearrangements. This suggests that iEκ^−/−^ pre-B cell rearrangements begin late enough in pro-B cell differentiation that only the most primary rearrangements take place, which are mostly Jκ1.

In summary, we have analyzed the unbiased gDNA and RNA repertoire of pro-B and pre-B cells and show that differences do occur in Vκ gene usage between the two libraries of a given cell type. These differences are likely tied to promoter strengths and appear consistent throughout B cell development. The overall distribution of Vκ gene rearrangements shifts toward Jκ-proximal Vκ gene usage during the course of BM B cell differentiation. Importantly, enhancer marks, especially H3K4me1, have the highest correlation with unequal Vκ utilization in pre-B cells, while PU.1 shows the highest correlation with early Vκ gene rearrangement in pro-B cells.

## Ethics statement

This study was carried out under approval of our protocol by The Scripps Research Institute's IACUC.

## Author contributions

EK and AF designed experiments, analyzed data, and wrote the manuscript. EK performed all experiments. SL performed the bioinformatic analyses.

### Conflict of interest statement

The authors declare that the research was conducted in the absence of any commercial or financial relationships that could be construed as a potential conflict of interest.
