# Supplementary figures and images for: Epigenetic Enhancer Marks and Transcription Factor Binding Influence Vκ Gene Rearrangement in Pre-B Cells and Pro-B Cells
Source: Front Immunol. 2018 Sep 13;9:2074. doi: 10.3389/fimmu.2018.02074 (PMC6146092; doi:10.3389/fimmu.2018.02074)

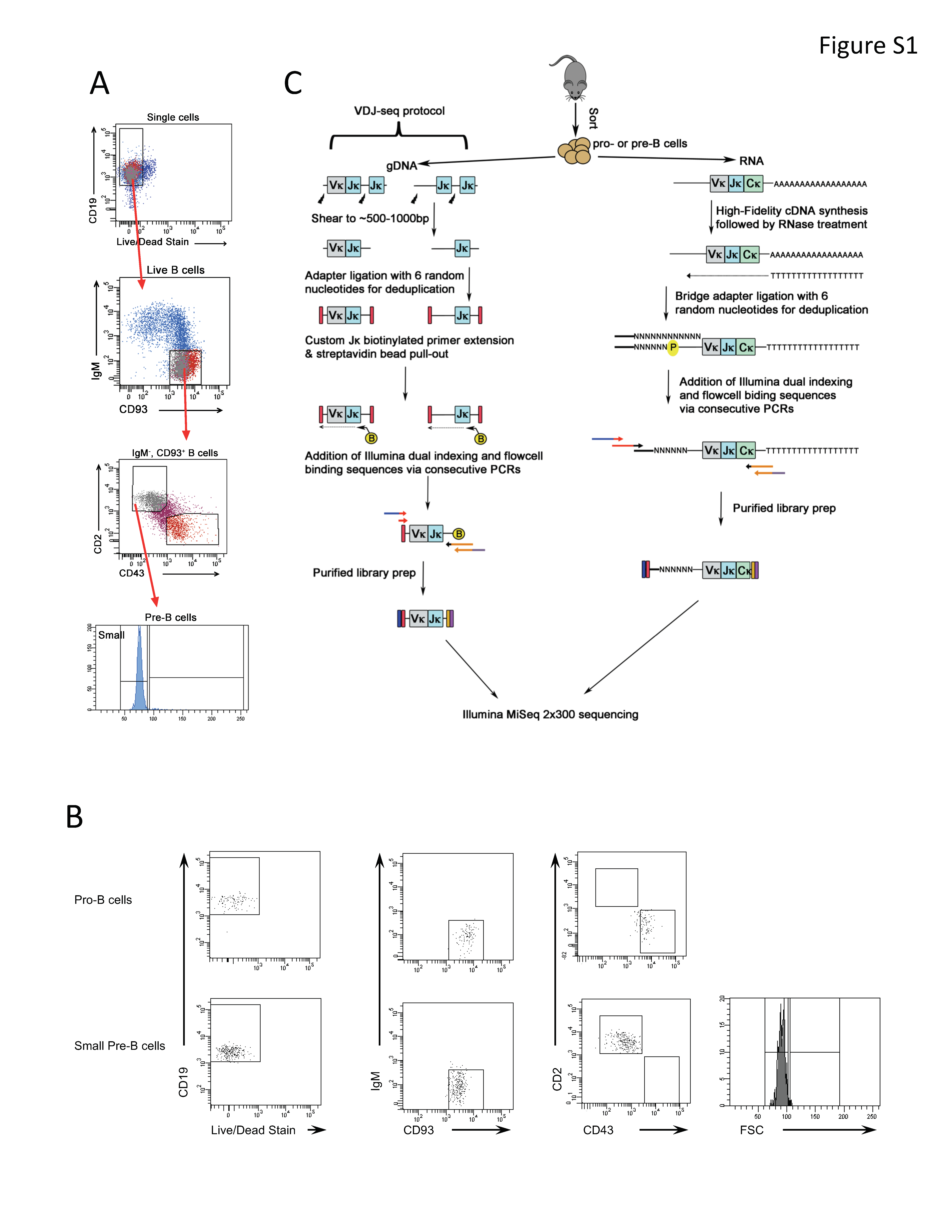

Supplement: Supplementary file 1 [file Image_1.TIF]

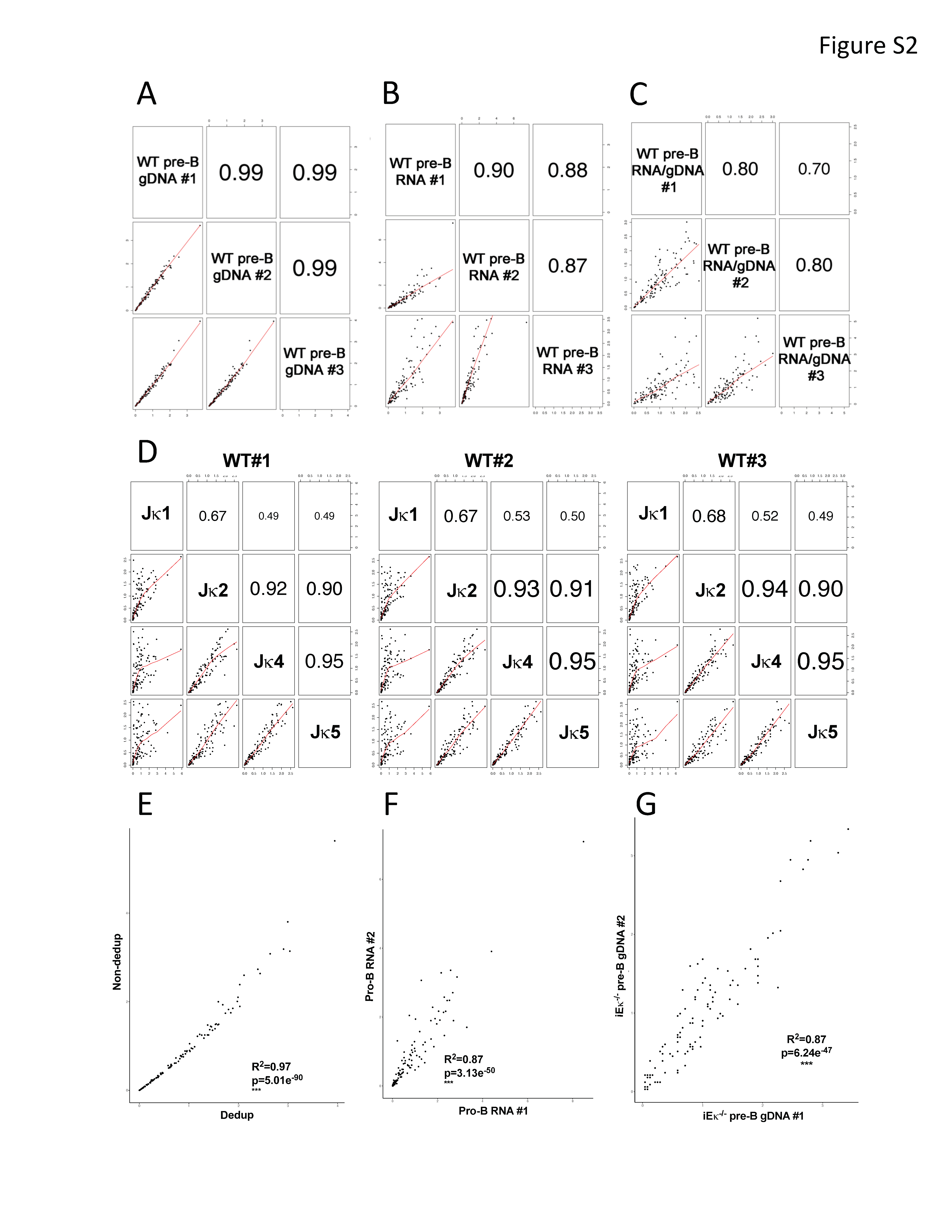

Supplement: Supplementary file 2 [file Image_2.TIF]

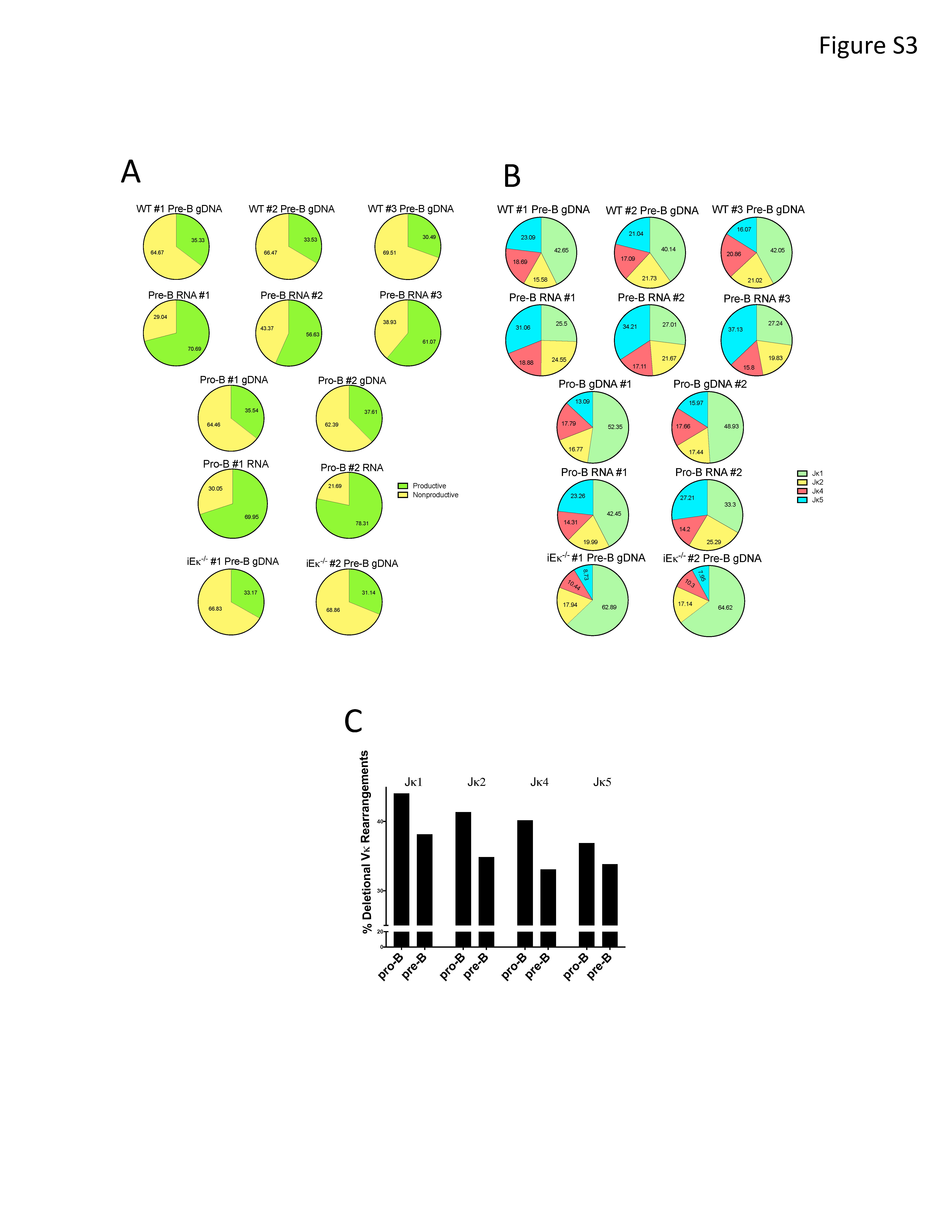

Supplement: Supplementary file 3 [file Image_3.TIF]

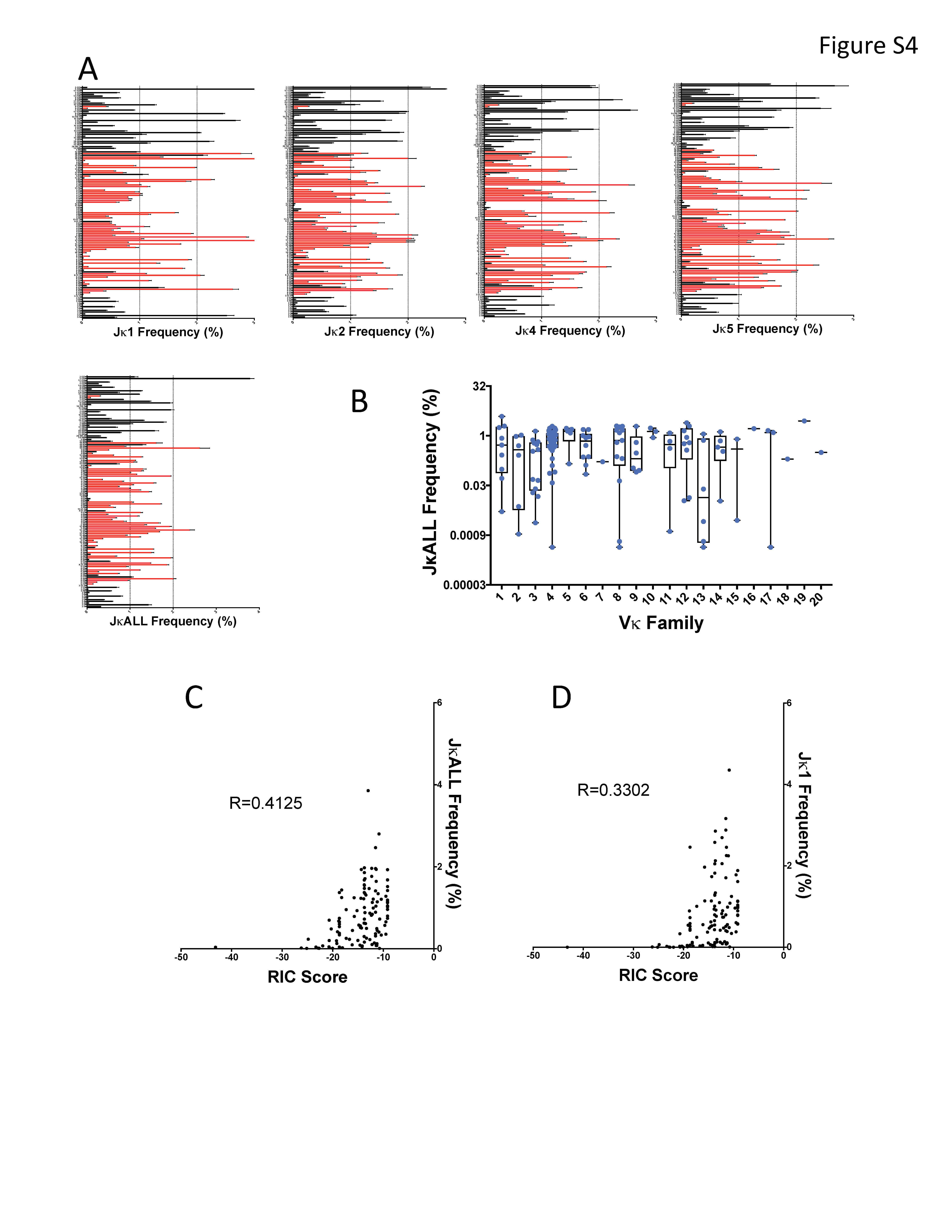

Supplement: Supplementary file 4 [file Image_4.TIF]

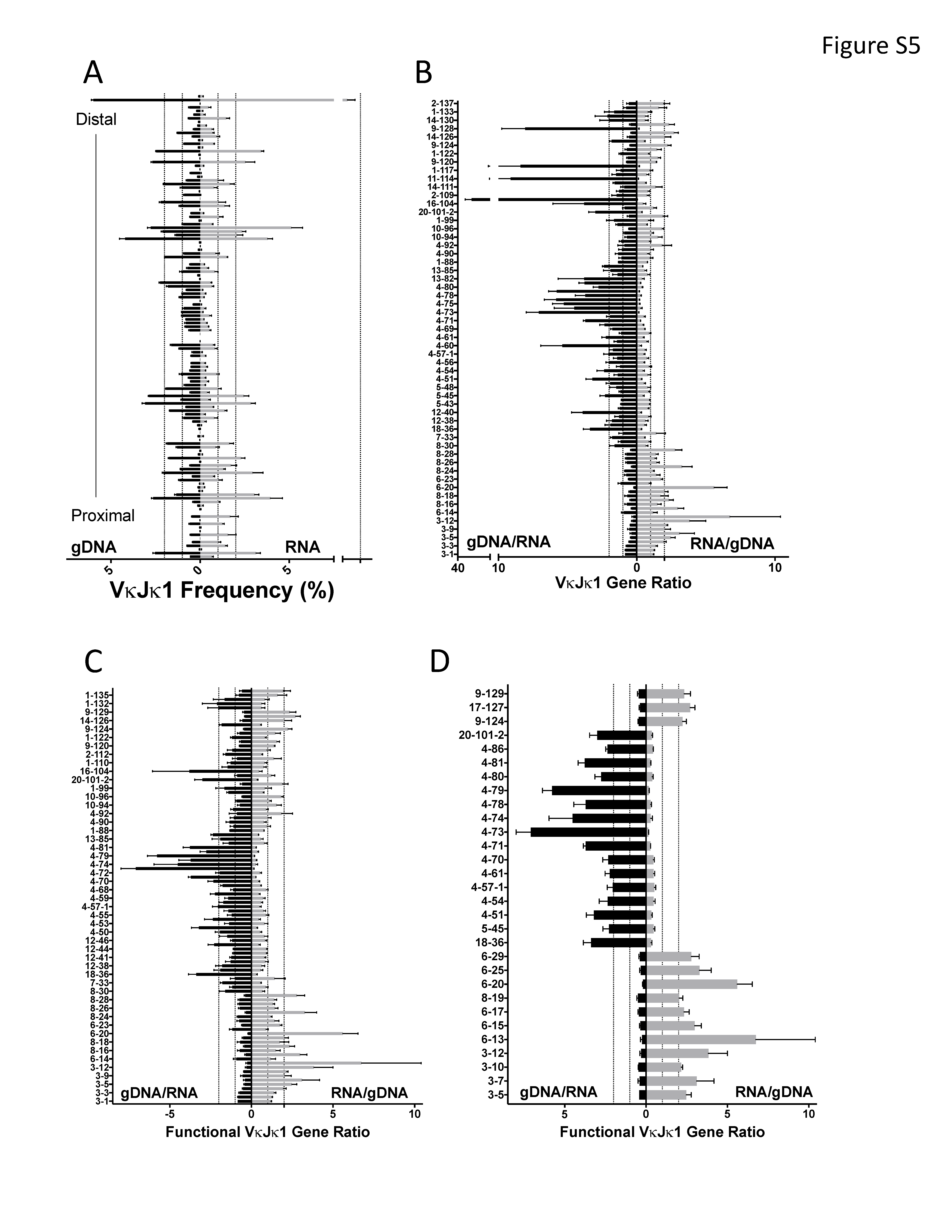

Supplement: Supplementary file 5 [file Image_5.TIF]

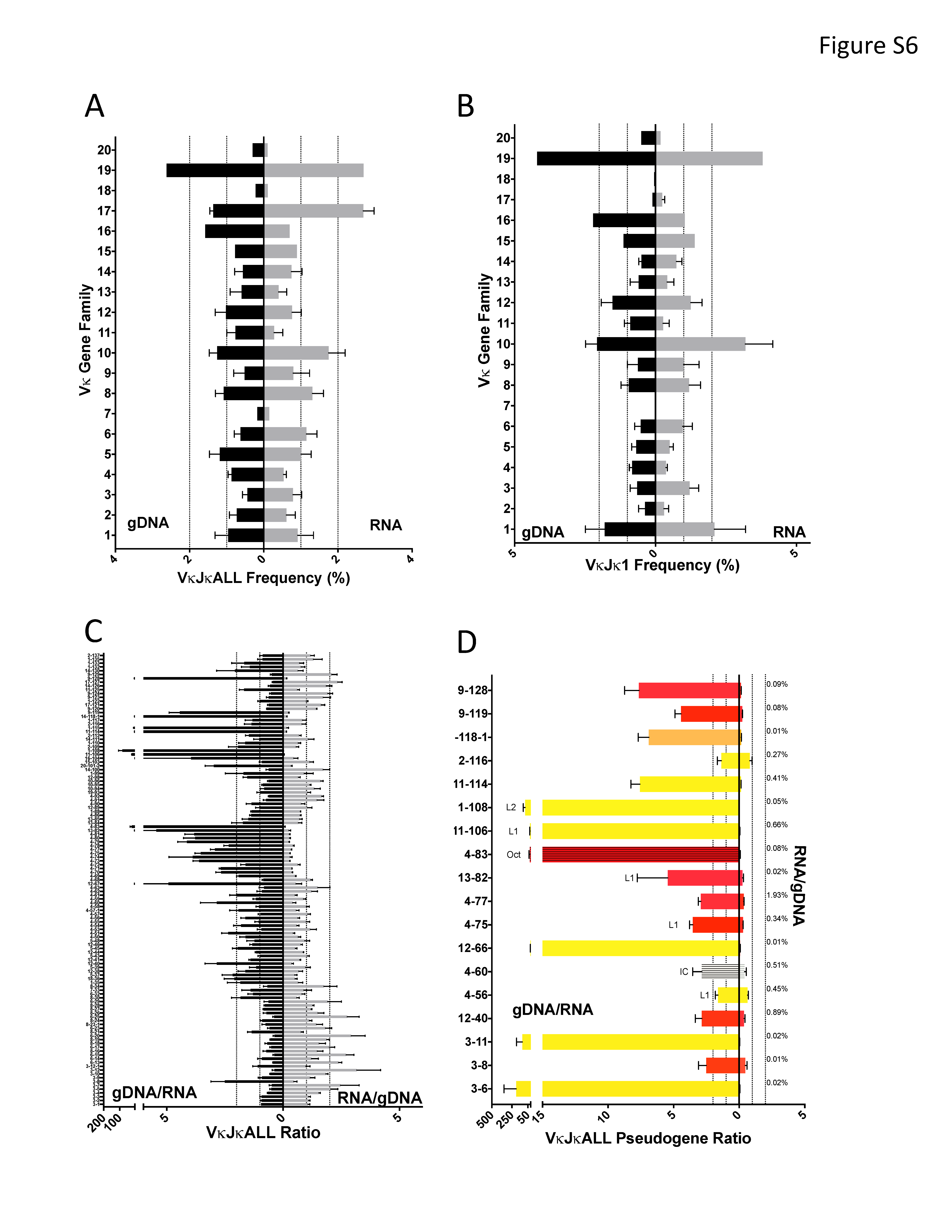

Supplement: Supplementary file 6 [file Image_6.TIF]

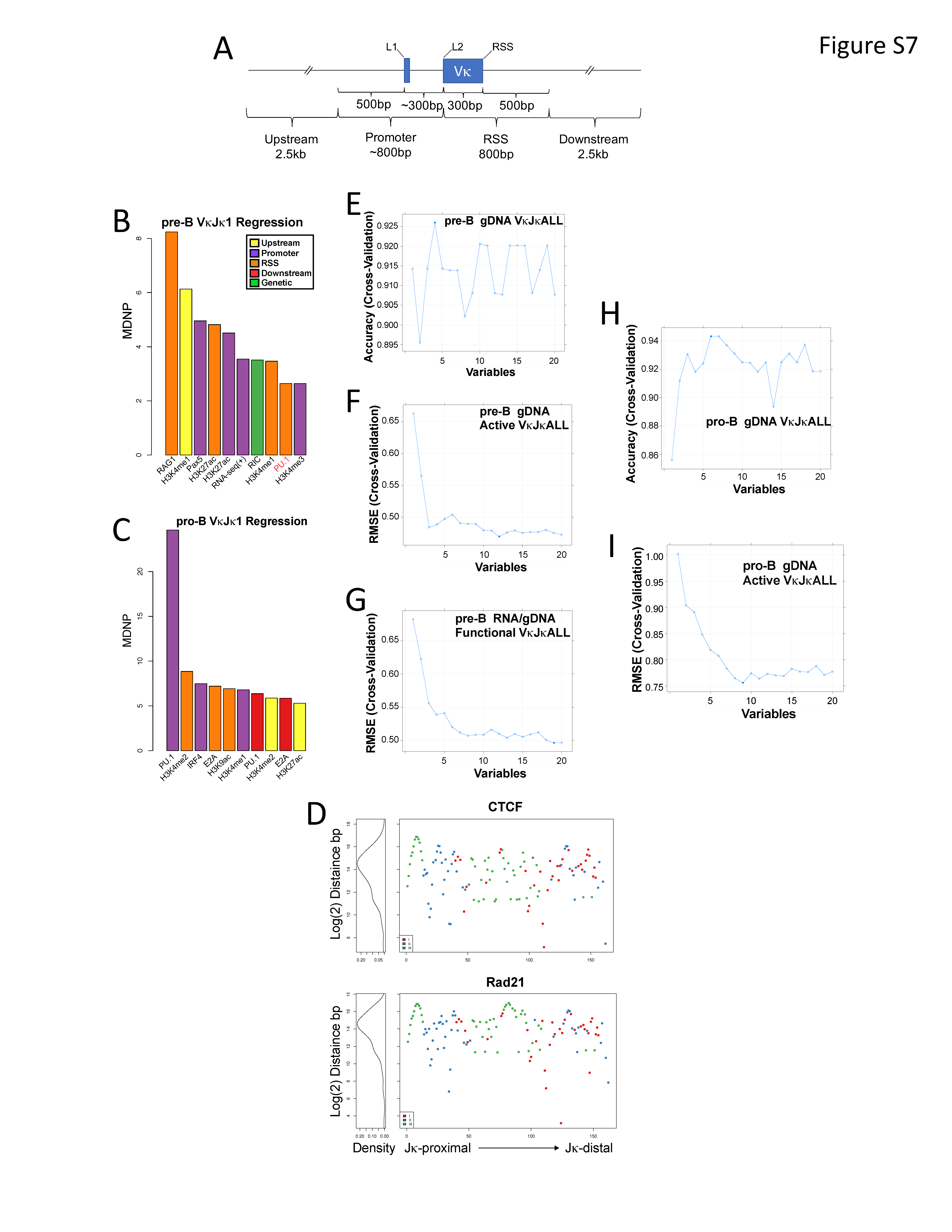

Supplement: Supplementary file 7 [file Image_7.TIF]

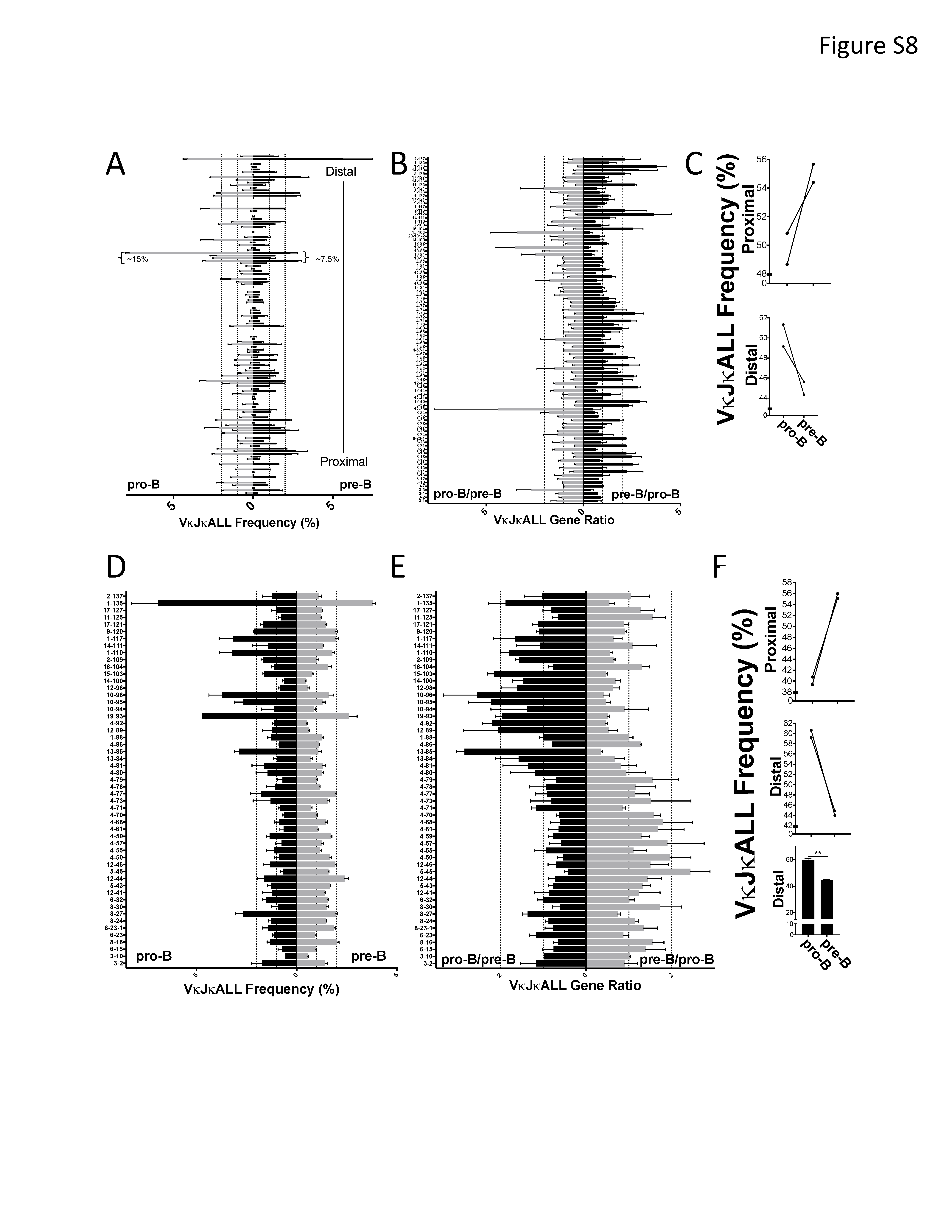

Supplement: Supplementary file 8 [file Image_8.TIF]

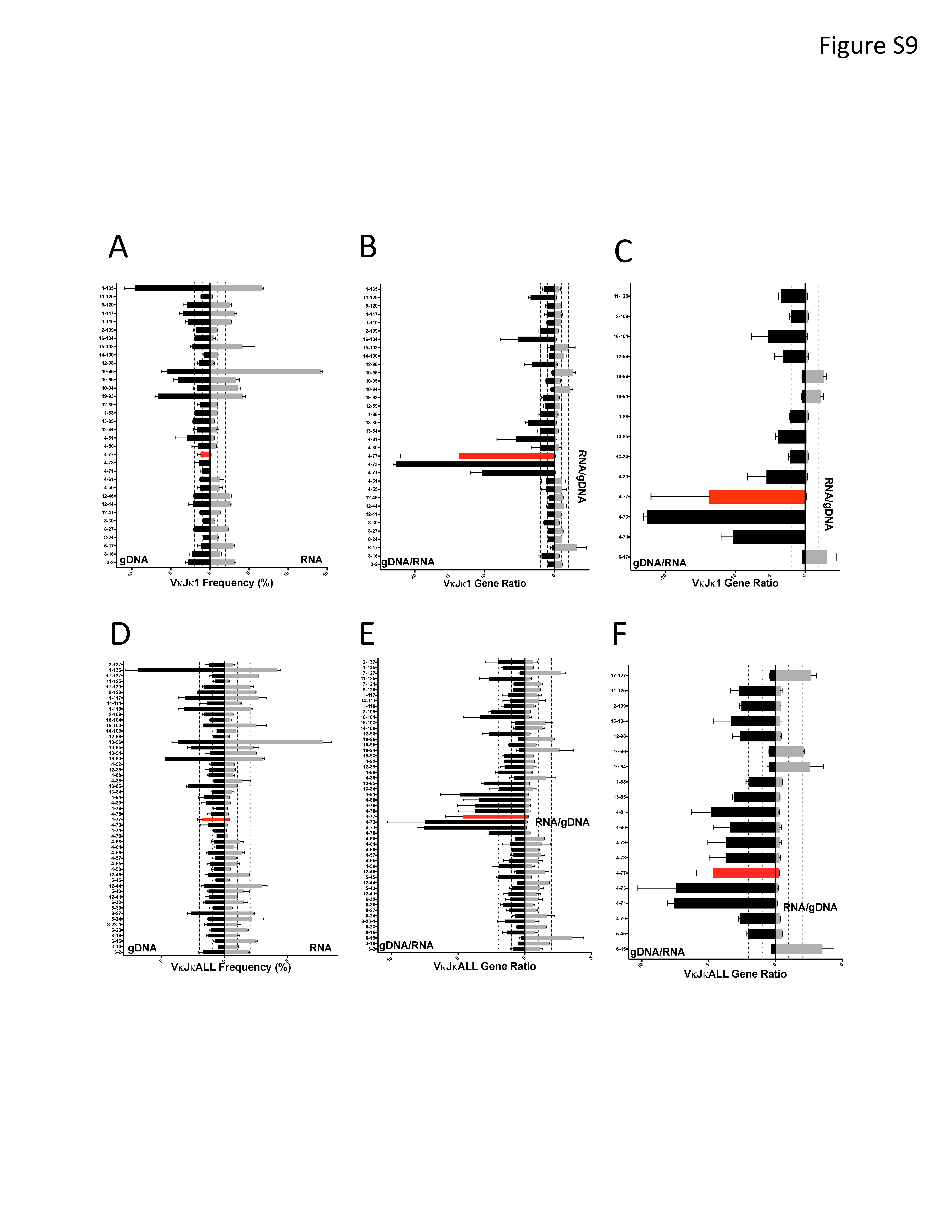

Supplement: Supplementary file 9 [file Image_9.TIF]

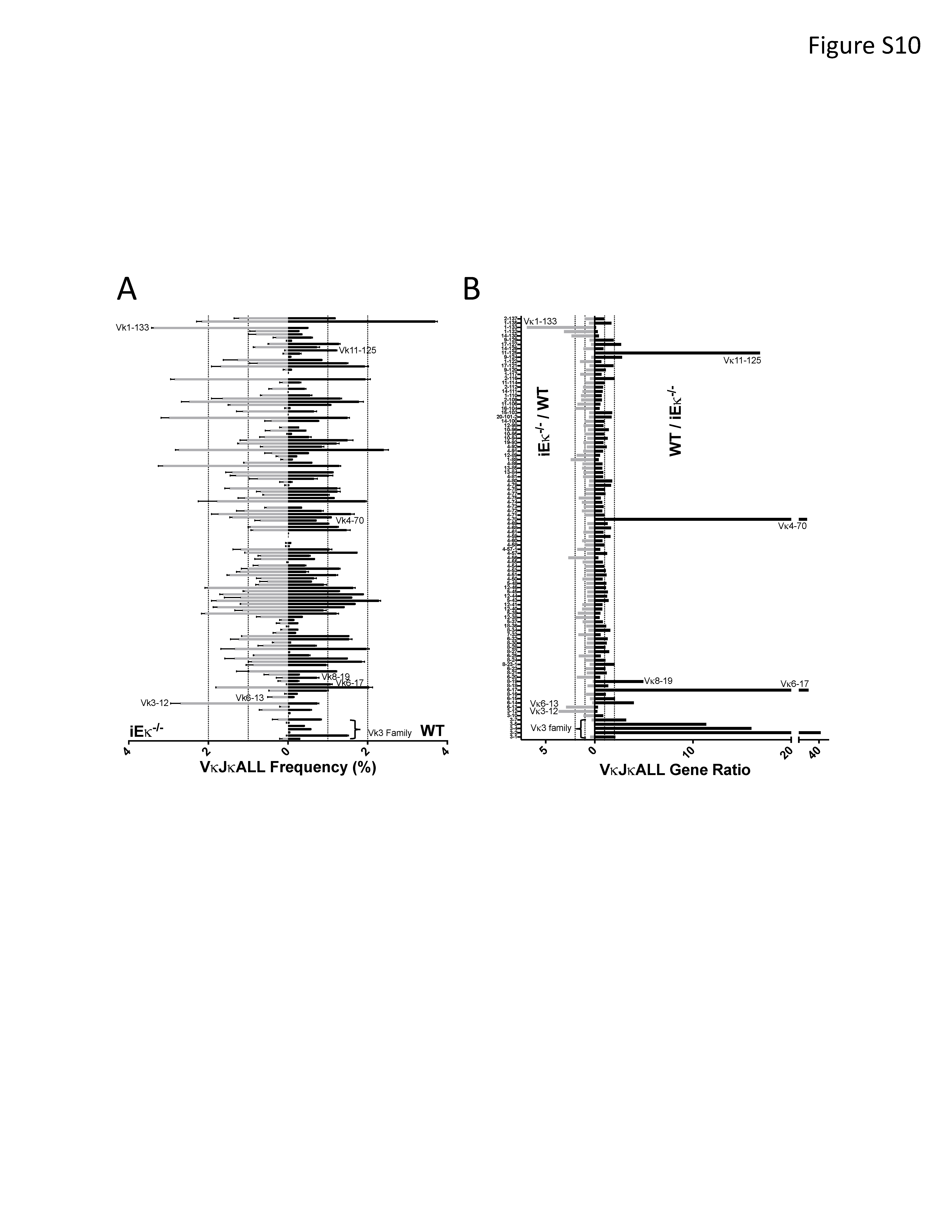

Supplement: Supplementary file 10 [file Image_10.TIF]
