# Supplementary material for: Epigenetic Enhancer Marks and Transcription Factor Binding Influence Vκ Gene Rearrangement in Pre-B Cells and Pro-B Cells
Source: Front Immunol. 2018 Sep 13;9:2074. doi: 10.3389/fimmu.2018.02074 (PMC6146092; doi:10.3389/fimmu.2018.02074)
Supplement: Supplementary file 11 [file Table_1.pdf]

Table S1. Feeney lab ChIP-seq and RNA-seq datasets used in this study

Feature	B-cell	Series	Accession
H3K4me1	Pro-B	GSE73531	GSM1897403
H3K4me1	Pre-B	GSE79054	GSM2084579
H3K4me3	Pro-B	GSE47766	GSM1156663
H3K4me3	Pre-B	GSE113306	GSM3103100
H3K27ac	Pre-B	GSE113306	GSM3103098
H3K27me3	Pro-B	GSE73531	GSM1897395
H3ac	Pro-B	GSE47766	GSM1156657
Pax5	Pre-B	GSE113306	GSM3103102
YY1	Pro-B	GSE73531	GSM1897387
YY1	Pre-B	GSE73531	GSM1897389
CTCF	Pro-B	GSE47766	GSM1156665
CTCF	Pre-B	GSE109909	GSM2973687
Rad21	Pro-B	GSE47766	GSM1156667
Rad21	Pre-B	GSE109909	GSM2973688
RNA-seq	Pro-B	GSE73532	GSM1897405, GSM1897406, GSM1897407
RNA-seq	Pre-B	GSE73532	GSM1897411, GSM1897412

Table S1. Publically available ChIP-seq datasets used in this study

Feature	B-cell	Reference	Series	Accession	Analysis and notes
H3K27ac	Pro-B + IL-7	[1]	GSE24165	GSM594592	
H3K9Ac	Pro-B + IL-7	[2]	GSE38046	GSM932943-6	Four replicates combined
H3K4me2	Pro-B + IL-7	[3]	GSE40173	GSM987804	
Pax5	Pro-B + IL-7	[2]	GSE38046	GSM932924	Bed file with all uniquely aligned reads
Ikaros	Pro-B + IL-7	[4]	GSE53595	GSM1296535	
Ikaros	Pre-B via IL-7	[5]	GSE42462	GSM1040573-4	Two replicates combined
IRF4	Pro-B + IL-7	[4]	GSE53595	GSM1296534	
E2A	Pro-B + IL-7	[6]	GSE21978	GSM546523	
EBF	Pro-B + IL-7	[4]	GSE53595	GSM1296532	
PU.1	Pro-B + IL-7	[4]	GSE53595	GSM1296533	
p300	Pro-B + IL-7	[3]	GSE40173	GSM987808	
Mediator1	Abl pro-B cell line	[7]	GSE44288	GSM1038263	
Foxo1	Pro-B + IL-7	[6]	GSE21978	GSM546525	Two runs combined (SRA)
Brg1	Pro-B + IL-7	[8]	GSE66978	GSM1635413	

Rag1	Pre-B (IgH Tg)	[9]	GSE69475	GSM1701796	

Table S1. VκJκ-seq datasets generated in this study

Feature	Series	Accession	Notes
WT pre-B cells gDNA #1	GSE117800	GSM3309446	
WT pre-B cells gDNA #2	GSE117800	GSM3309447	
WT pre-B cells gDNA #3	GSE117800	GSM3309448	
WT pre-B cells RNA #1	GSE117800	GSM3309449	Technical replicates combined
WT pre-B cells RNA #2	GSE117800	GSM3309450	
WT pre-B cells RNA #3	GSE117800	GSM3309451	
WT pro-B cells gDNA #1	GSE117800	GSM3309452	
WT pro-B cells gDNA #2	GSE117800	GSM3309453	
WT pro-B cells RNA #1	GSE117800	GSM3309454	
WT pro-B cells RNA #2	GSE117800	GSM3309455	
iEκ$^{-/-}$ pre-B cells gDNA #1	GSE117800	GSM3309456	Technical replicates combined
iEκ$^{-/-}$ pre-B cells gDNA #2	GSE117800	GSM3309457	Technical replicates combined

References

1. Creyghton, M.P., et al., *Histone H3K27ac separates active from poised enhancers and predicts developmental state.* Proc Natl Acad Sci U S A, 2010. **107**(50): p. 21931-6.
2. Revilla, I.D.R., et al., *The B-cell identity factor Pax5 regulates distinct transcriptional programmes in early and late B lymphopoiesis.* EMBO J, 2012. **31**(14): p. 3130-46.
3. Lin, Y.C., et al., *Global changes in the nuclear positioning of genes and intra- and interdomain genomic interactions that orchestrate B cell fate.* Nat Immunol, 2012. **13**(12): p. 1196-204.
4. Schwickert, T.A., et al., *Stage-specific control of early B cell development by the transcription factor Ikaros.* Nat Immunol, 2014. **15**(3): p. 283-93.
5. Ferreiros-Vidal, I., et al., *Genome-wide identification of Ikaros targets elucidates its contribution to mouse B-cell lineage specification and pre-B-cell differentiation.* Blood, 2013. **121**(10): p. 1769-82.
6. Lin, Y.C., et al., *A global network of transcription factors, involving E2A, EBF1 and Foxo1, that orchestrates B cell fate.* Nat Immunol, 2010. **11**(7): p. 635-43.
7. Whyte, W.A., et al., *Master transcription factors and mediator establish super-enhancers at key cell identity genes.* Cell, 2013. **153**(2): p. 307-19.
8. Bossen, C., et al., *The chromatin remodeler Brg1 activates enhancer repertoires to establish B cell identity and modulate cell growth.* Nat Immunol, 2015. **16**(7): p. 775-84.
9. Teng, G., et al., *RAG Represents a Widespread Threat to the Lymphocyte Genome.* Cell, 2015. **162**(4): p. 751-65.
