# Supplementary material for: Epigenetic Enhancer Marks and Transcription Factor Binding Influence Vκ Gene Rearrangement in Pre-B Cells and Pro-B Cells
Source: Front Immunol. 2018 Sep 13;9:2074. doi: 10.3389/fimmu.2018.02074 (PMC6146092; doi:10.3389/fimmu.2018.02074)
Supplement: Supplementary file 14 [file Data_Sheet_3.docx]

**Supplementary Figure Legends**

**Figure S1.  Pro-B and small pre-B sorting scheme and MiSeq library prep**.  (A) CD19^+^ microbead purified BM cells were gated for live CD19^+^ B cells and further gated for CD93^high^, IgM^-^ expression.  Cells were then gated as either pro-B cells (CD43^+^, CD2^-^) or pre-B cells (CD43^-^, CD2^+^).  Pre-B cells were further gated based on size.  Cells on the right side of the forward scatter histogram were large pre-B cells.  Cells on the left side were small pre-B cells.  Only small pre-B cells were used in this study.  (B) Post-sort purity assessment from the lymphocyte gate for pro-B cells (80% pure, upper panels) and small pre-B cells (98.7% pure, lower panels). Small pre-B cell post-sort analysis also includes FSC histogram. Sort gates were kept the same for the post sort assessment, and loss of BV421signal intensity (CD43) is apparent for both B cell populations after sort, accounting for the apparent loss in purity in pro-B cells. (C) gDNA or RNA was harvested from sorted cells.  For gDNA, VDJ-seq library preparation was performed as recently described by Matheson et al. with modifications (left side). Briefly, gDNA was sheared to a size of 500-1000 bp using Bioruptor (Diagenode).  Sheared gDNA was then adapter ligated.  This adapter had 6N for deduplication.  Adapter ligated gDNA was primer extended using combination of custom Jκ1, Jκ2, Jκ4, and Jκ5 biotinylated primers followed by streptavidin bead pull-out.  The first of two PCRs adds the Illumina i5 and i7 index primer sequence to the biotinylated primer extended DNA.  The 3’ end of the reverse primer is positioned just upstream of the binding site for the biotinylated Jκ primer.  The second PCR adds the P5 and P7 sequences to complete the gDNA library prep.  We developed a custom protocol for RNA-based library prep (right side).   RNA is converted to cDNA using a high-fidelity (HF) reverse transcriptase (RT).  This is followed by RNase H, then RNase A/T treatment to eliminate any contaminating RNA.  Purified cDNA is then adapter ligated using “bridge” adapters with custom 6 random Ns for deduplication.  The 6N serving as a bridge will not be incorporated into the amplified sequence.  The 6Ns containing the phosphate will be incorporated into the amplified sequence.  As was the case for gDNA library prep, consecutive PCRs add the i5, i7 and P5, P7 sequences.  Both gDNA and cDNA library preps were sequenced using Illumina MiSeq 2x300 read lengths.

**Figure S2. Repertoire correlations**. (A-D) Scatterplot matrices (lower left panels) and absolute correlation (upper right panels) were generated using the pairs and panel.cor functions, respectively, in R.  Scatterplot matrices and correlation for (A) 3 pre-B gDNA biological replicates, (B) 3 pre-B RNA biological replicates or (C) the rearrangement frequency ratios of the 3 gDNA/RNA pairs, where each pair were from the same sorted cells.  VκJκALL gene frequency was used as input for A-C.  The font size of the correlation is proportional to the correlation. A-C, Vκ genes with no reads in all 3 replicates were excluded from analysis.  (D) Scatterplot matrices and correlation of individual Jκ repertoires for each pre-B cell gDNA biological replicate are shown.  WT#3 library prep was generated using different Jκ reverse PCR primers. The font size of the correlation is proportional to the actual correlation.  (E-G) Scatterplots using ggplot package in R. Correlation and p value were generated using gtools package in R.  (E) Correlation between pre-B cell RNA sample with (Dedup) and without (Non-dedup) deduplication based on the adapter random 6Ns.  (F) Correlation between pro-B cell RNA biological replicates.  (G) Correlation between iEκ^-/-^ pre-B cell gDNA biological replicates.  E-G, samples used VκJκALL gene frequency as input.  Genes with 0% frequency in both samples of a given comparison were not included.

**Figure S3. Analysis of productive, non-productive, Jκ gene repertoire and V**κ **orientation repertoire**.  (A) Pie charts showing the percent of productive (green) versus non-productive (yellow) VκJκALL gene rearrangement frequency. Top row shows the 3 pre-B cells gDNA biological replicates. The second row shows the 3 paired pre-B cell RNA biological replicates.  Third row shows the 2 pro-B cell gDNA biological replicates from the same BM used to derive pre-B cell gDNA/RNA #1 and #2.  Fourth row shows the 2 pro-B cell RNA biological replicates from the same BM used to derive pre-B cell gDNA/RNA #1 and #2.  Bottom row shows the 2 iEκ^-/-^ pre-B cell gDNA biological replicates.  (B) Pie charts showing the percent usage of each Jκ gene.  Jκ1 (green), Jκ2 (yellow), Jκ4 (red) and Jκ5 (blue) gene usage are illustrated for each sample along with the percent usage.  Samples arranged as in A.  WT#3 pre-B gDNA library prep utilized different Jκ PCR reverse primers as described in Materials and Methods. (C) Percent gDNA rearrangements of deletional oriented Vκ genes for each Jκ gene from sorted pro-B and pre-B cells.  Percentage for pro-B cell rearrangements are based on the total reads from 2 biological replicates.  Percentage for pre-B cell rearrangements are based on the total reads from 3 biological replicates.

**Figure S4. Pre-B cell VκJκ** **rearrangement frequencies and relationship to RSS quality score (RIC score)**.  (A) Vκ gene rearrangement frequencies arranged from Jκ-proximal (bottom) to Jκ-distal (top) of 3 independent pre-B cell gDNA biological replicates. Black bars depict deletional rearrangements while red bars depict inversional rearrangements.  The only genes excluded are those that had 0% rearrangement frequency in VκJκALL for all 3 replicates.  Repertoire graphs are arranged in the following order from top left; VκJκ1, VκJκ2, VκJκ4, VκJκ5 and VκJκALL (below VκJκ1). Dotted vertical lines are arranged at 1% and 2 % rearrangement frequency.  Error bars represent SEM.  (B) Box and whisker graph of gDNA VκJκALL rearrangement frequencies grouped by Vκ gene family.  Each blue dot represents a Vκ gene within its respective gene family.  The box for each Vκ gene family represents the 25th to 75th percentile rearrangement frequency. The line in the middle of each box represents the median rearrangement frequency. The whiskers for each Vκ gene family box extend from the minimum to the maximum rearrangement frequency. (C-D) Correlation between (C) VκJκALL or (D) VκJκ1 repertoires (at a frequency of 0.01% of greater) to RIC scores. R Pearson correlation value depicts how gene rearrangement frequency correlates with RIC score.

**Figure S5. Comparison of pre-B cell VκJκ1 gDNA to RNA repertoires** (A) VκJκ1 gDNA frequencies (left side) versus VκJκ1 RNA frequencies (right side) plotted in descending order from Jκ-distal (top) to Jκ-proximal (bottom).  Dotted vertical lines depict 1%, 2% and 15% frequencies.  Only genes that had no reads in both gDNA and RNA repertoires are excluded. (B) VκJκ1 gene (functional and pseudogene) rearrangement frequency ratio of gDNA/RNA (left side) or the reciprocal RNA/gDNA (right side) are arranged vertically as in A. Dotted vertical lines depict 1 (no difference) and 2-fold changes. Only Vκ genes that had reads in all 3 biological replicates for both gDNA and RNA are included.  (C) VκJκ1 gene ratio for IMGT-designated functional genes arranged as in B.   (D) Functional Vκ genes that are at least 2-fold greater in gDNA (left) or 2-fold greater in RNA (right) and at least 1.5-fold greater in each paired replicate comparison.  C and D, each pairing of gDNA and RNA derives from the same sorted pre-B cells. Errors bars represent SEM.

**Figure S6. Comparison of pre-B cell gDNA to RNA relative rearrangement frequency**.  (A) VκJκALL or (B) VκJκ1 repertoire grouped by Vκ gene family.  gDNA is on the left side.  RNA is on the right side. Gene families are arranged numerically from 1 (bottom) to 20 (top). (C) VκJκALL gene (functional and pseudogenes) repertoire ratios of gDNA/RNA (left side) or the reciprocal RNA/gDNA (right side) for all 3 biological replicates combined are arranged vertically from Jκ-distal (top) to Jκ-proximal (bottom).  Dotted vertical lines depict 1 (no difference) and 2-fold changes. Only genes with reads in all 3 biological replicates for both gDNA and RNA are included.  (D) VκJκALL rearrangement frequency ratios for all IMGT-classified pseudogenes comparing gDNA/RNA (left) versus RNA/gDNA (right) from all 3 paired biological replicates.  Vκ14-126 was excluded from the pseudogene list as it is really a functional gene according to IMGT.  In addition, Vκ1-35 and Vκ8-18 are also omitted as they are classified as open reading frame genes in IMGT.  The VκJκALL gDNA rearrangement frequency (percentage) for each pseudogene is listed on the right side.  Red bars indicate the presence of STOP codons, yellow bars indicate frameshift mutations, deletions and insertions, orange bar indicates a combination of stop-codons and frameshift mutations, horizontal stripes indicate lack of an initiation codon.  Pseudogenes whose defect occurs near the beginning of the gene are indicated by the location of the defect as either Leader-part 1 (L1), Leader-part 2 (L2), Initiation Codon (IC) or Octamer (Oct). Only genes which averaged at least 0.01% in the 3 gDNA replicate samples are shown.  In several instances, RNA samples that had no reads were assigned one read to be able to derive ratios.  C and D, each pairing of gDNA and RNA derives from the same sorted pool of pre-B cells.   A-D, genes with 0% frequency in both RNA and gDNA are excluded.  Errors bars represent SEM.

**Figure S7. VκJκ1 RF regression, minimum distance from Vκ gene to CTCF/Rad21 and Recursive Feature Elimination analysis**.  (A) Schematic of four non-overlapping windows used in RF analysis.  Example shown is for a Vκ gene in the deletional orientation.  Promoter window extends 500 bp upstream of leader 1 start and extends ~300 bp downstream of the leader 1 start.  Due to the slight variability in the intronic sequence length separating leader 1 and leader 2, the promoter window will be slightly different for Vκ genes.  The RSS window extends 300 bp upstream of the RSS site encompassing the coding region and 500 bp downstream of the end of the coding region.  The upstream window extends 2500 bp upstream of the promoter window.  The downstream window extends 2500 bp downstream of the RSS window. (B, C) Variable importance for each chromatin and RNA feature in a RF regression model for rearrangement frequency in (B) pre-B cell or (C) pro-B cell VκJκ1 active genes. Shown are features with significant variable importance. Mean Decrease in Node Purity (MDNP) is a measure of the decrease in accuracy if the feature is excluded. Chromatin or RNA feature is listed below bar.  Features in A derived from pro-B cells are indicated in red while features in black are from pre-B cells.  All features used in B are from pro-B cells.  (D) Distance to closest CTCF (upper scatterplot) or Rad21 (lower scatterplot) binding sites of individual Vκ genes arranged from Jκ-proximal (left) to Jκ-distal (right). Distance to closest CTCF and Rad21 sites is plotted in log2 bp.  A Vκ gene (circle) located close to a CTCF/Rad21 site will appear at the bottom of the graph whereas a Vκ gene far from CTCF/Rad21 site will appear at the top of the graph.  Genes are colored according to the IMGT designated clans they belong to.  Genes belonging to clan I are red, clan II are blue, and clan III are green. A histogram of the density of genes at each distance is shown vertically on the left. (E-I) Recursive Feature Elimination (RFE) analysis using the top 20 features from variable importance/classification analysis as input. (E) RFE of inactive and active VκJκALL genes from pre-B cell gDNA using the top 20 features from the classification analysis (Figure 3A). The y axis is a measure of model accuracy with a higher number indicating more accuracy. The x axis denotes the number of variables needed to achieve optimal accuracy. The 4 factors which together give the highest accuracy (~0.925) are RIC score, Ikaros_pre-B_RSS, PU.1_pro-B_RSS and Ikaros_pre-B_Promoter. (F) RFE analysis of active VκJκALL genes from pre-B cell gDNA using the top 20 features from the variable importance regression analysis (Figure 3B). Root Mean Square Error (RMSE) is a measure of accuracy with a lower number signifying more accuracy. The x axis denotes the number of variables needed to achieve optimal accuracy. The 3 factors which together contribute the most to a low RMSE (~0.475) are H3K4me1_pre-B_RSS, and PU.1_pro-B_RSS, and RIC score. (G) RFE analysis of functional VκJκALL RNA/gDNA pre-B cell gene ratios using the top 20 features from the variable importance regression analysis (Figure 3E). The 6 factors which together contribute most to a low RMSE (~0.52) are H3K4me1_pre-B_Promoter, Ikaros_pre-B_Promoter, EBF_pro-B_Promoter, PU.1_pro-B_Promoter, H3K9ac_Upstream, and E2A_Promoter. (H) RFE analysis of inactive and active VκJκALL genes from pro-B cell gDNA using the top 20 features from the classification analysis (Figure 6A). The 3 factors which contribute most to optimal accuracy (~0.93) are RIC score, PU.1_RSS, and H3K4me1_RSS. (I) RFE analysis of active VκJκALL genes from pro-B cell gDNA using the top 20 features from the regression analysis (Figure 6B). The 9 features that give the most accuracy (~0.75) are PU.1_RSS, H3K4me2_Downstream, YY1_Upstream, E2A_RSS, Ikaros_Downstream, H3K9ac_Promoter, PU.1_Promoter, H3K4me1_Promoter, and CTCF_RSS.

**Figure S8. Pro-B cell VκJκALL repertoire is biased to Jκ-distal Vκ genes**.  (A) VκJκALL RNA repertoire frequencies of pro-B cells (left) and pre-B cells (right) arranged from Jκ-distal (top) to Jκ-proximal (bottom).  Dotted vertical lines represent 1% and 2% rearrangement frequencies.  Brackets encompass Vκ genes Vκ19-93 to Vκ10-96.  Only genes with at least one read in either sample are shown. (B) VκJκALL RNA rearrangement frequency ratios of pro-B/pre-B (left) and pre-B/pro-B (right) arranged vertically as in A.  Dotted vertical lines represent 1 (no difference) and 2-fold changes in RNA ratio.  Only Vκ genes where both pro-B cell replicates had 0.05% frequency or greater are shown. Error bars represent SEM.  (C) Comparison of pro-B and pre-B cell VκJκALL RNA frequency in the Jκ-distal versus Jκ-proximal kappa locus region. Jκ-proximal Vκ genes include genes Vκ3-1 to Vκ13-76 (top bar graph).  Jκ-distal Vκ genes include Vκ4-77 to Vκ2-137 (bottom bar graph). Lines connect pro-B and pre-B cells from the same sorted mice. (D) VκJκALL gDNA repertoire frequencies of pro-B (left) and pre-B cells (right) arranged from Jκ-distal (top) to Jκ-proximal (bottom).  Dotted vertical lines represent 1% and 2% rearrangement frequencies. Only Vκ genes with at least 0.5% in both pro-B replicates were included. (E) VκJκALL gDNA rearrangement frequency ratios of pro-B/pre-B (left) and pre-B/pro-B (right) arranged vertically as in D.  Dotted vertical lines represent 1 (no difference) and 2-fold changes in relative gDNA rearrangement. (F) Comparison of pro-B and pre-B cell VκJκALL gDNA rearrangements in the Jκ-distal versus Jκ-proximal kappa locus region as in C.  Bottom plot compares Jκ-distal rearrangement of both replicates combined for either pro-B or pre-B cells. Error bars represent SEM.  ** represents a p value < 0.01.

**Figure S9. Repertoire gDNA and RNA ratios of pro-B cells**.  (A) Pro-B cell VκJκ1 gDNA frequencies (left side) versus pro-B cell VκJκ1 RNA repertoire frequencies (right side) plotted in descending order from Jκ-distal (top) to Jκ-proximal (bottom).  Dotted vertical lines depict 1% and 2% frequencies.  Vκ genes with at least 0.5% frequency in both pro-B cell gDNA replicate samples are included.  Vκ4-77 is a pseudogene with a stop codon and is marked by a red bar.  (B) Pro-B cell VκJκ1 gDNA/RNA ratio (left side) or reciprocal RNA/gDNA rearrangement frequency ratios (right side) are arranged vertically as in A.  Dotted vertical lines depict 1 (no difference) and 2-fold changes in either direction.  (C) Subset list from B of Vκ genes that were at least 2-fold greater in gDNA (left) or at least 2-fold greater in RNA (right) and at least 1.5-fold greater in each paired comparison.  For B and C, ratios are calculated by pairing gDNA and RNA from the same sorted pool of pro-B cells, 2 biological replicates total.  (D-F) VκJκALL data arranged as in A-C.  Errors bars represent SEM.

**Figure S10. iEκ regulates Vκ3 family gene pre-B cell rearrangements**.  (A) VκJκALL gene gDNA rearrangement frequency in WT (right) and iEκ^-/-^ (left) pre-B cells.  Labeled genes indicate differentially rearranged Vκ genes. Only Vκ genes that had reads in either WT or iEκ^-/-^ cells are listed.  (B) VκJκALL gDNA repertoire ratios comparing WT and iEκ^-/-^ pre-B cells.  Left side bars show the iEκ^-/-^/WT ratio.  Right side shows the WT/iEκ^-/-^ ratio.  Only VκJκALL genes that are at least 0.1% in WT are listed.  iEκ^-/-^ genes which had a rearrangement frequency of 0 (when paired with WT genes that had a frequency ≥ 0.1) were replaced with the frequency equivalent of 1 read.  Therefore, some of the WT/iEκ^-/-^ ratios above 1-fold are an underrepresentation of the actual difference between the two. Data for A and B are comprised of 2 WT replicates and 2 iEκ^-/-^ replicates.  For B, the replicates of WT and iEκ^-/-^ were combined to produce one value for a Vκ gene ratio. All error bars represent SEM.
